# Cast versus functional brace in the rehabilitation of patients treated for an ankle fracture: protocol for the UK study of ankle injury rehabilitation (AIR) multicentre randomised trial

**DOI:** 10.1136/bmjopen-2018-027242

**Published:** 2018-12-18

**Authors:** Rebecca Samantha Kearney, Rebecca McKeown, Siobhan Stevens, Nicholas Parsons, Helen Parsons, Philip Wells, Jaclyn Brown, Martin Underwood, Anthony Redmond, James Mason, Matthew L Costa

**Affiliations:** 1 Warwick Medical School, University of Warwick, Coventry, UK; 2 Leeds Institute of Rheumatology and Musculoskeletal Medicine, University of Leeds, Chapel Allerton Hospital, Leeds, UK; 3 Orthopaedic Trauma, Oxford University, John Radcliffe Hospital, Oxford, UK

**Keywords:** rehabilitation medicine, adult orthopaedics

## Abstract

**Introduction:**

Each year in the UK over 120 000 people fracture their ankle. It is not known what the best rehabilitation strategy is for these people. Traditionally standard care has involved immobilisation in a plaster cast but an alternative is a functional brace, which can be removed to allow early movement. This paper details the protocol for a multicentre randomised trial of plaster cast immobilisation versus functional bracing for patients with an ankle fracture.

**Methods and analysis:**

We will recruit adults with a fractured ankle, for which the treating clinician would consider plaster cast to be a reasonable management option. Randomisation will be on a 1:1 basis, stratified by centre, operative or non-operative management and age. Participants will be allocated to either plaster cast or a functional brace, both treatments are widely used. To have 90% power to detect a difference of 10 points on the primary outcome (Olerud and Molander Ankle Score) at the primary outcome time point (16 weeks), we need to randomise a minimum of 478 people. Quality of life and resource use will be collected at 6, 10, 16, 24 weeks and 12, 18, 24 months. The differences between treatment groups will be assessed on an intention-to-treat basis. The economic evaluation will adhere to the recommendations of the National Institute for Health and Care Excellence reference case.

**Ethics, registration and dissemination:**

National Research Ethic Committee approved this study on 4 July 2017 (17/WM/0239). The first site opened to recruitment 9 October 2017. The results of this trial will be submitted to a peer-reviewed journal and will inform clinical practice.

**Trial registration number:**

ISRCTN15537280; Pre-results.

**Strengths and limitations of this study**

- Broad eligibility criteria to ensure results can be generalised to the wider population.
- UK-wide trial across a minimum of 19 centres to optimise external validity.
- Primary outcome measure is patient centred.
- Blinding of interventions is not possible.

## Introduction

Nine per cent of trauma surgeons’ workload in the UK is managing ankle fractures; over 120 000 per year in the UK. A threefold increase is expected by 2030 due to an increase in older adults who are remaining physically active.^1 2^ The frequency of this injury is an increasing burden on the National Health Service (NHS) year on year.^3^ The short-term impact of this injury results in physical impairments of pain, stiffness, weakness and swelling. The longer term impact results in extended time off work and development of post-traumatic arthritis.^4 5^

Management has traditionally included plaster cast immobilisation for several weeks, while the bone heals. A cast provides maximum support, however, there are potential problems, there are the risks associated with prolonged immobilisation such as muscle atrophy, deep vein thrombosis and joint stiffness. There are the long-term consequences, which include prolonged gait abnormalities, persistent calf muscle weakness and an inability to return to previous activity levels. Functional bracing may address these issues.^6^ However, it does not provide the same degree of support to the healing bones.^5 7^

A research priority exercise was undertaken by a UK orthopaedic group in 2010.^8^ One of the top priority questions was to establish whether there is a functional advantage associated with different rehabilitation plans following an ankle fracture. This was followed by a 2012 Cochrane review^9^ which identified the need for further research into the optimal method of immobilisation for both operatively and non-operatively managed acute ankle fractures. This issue was raised again in a James Lind Alliance Priority Setting Partnership in 2018, which included: ‘What is the best physiotherapy regimen for adults during out-of-hospital recovery from a fragility fracture of the lower limb?’ in its top 10 priority research questions.^10^

## Good Clinical Practice

The trial will be conducted in full conformance with the principles of the Declaration of Helsinki and to Good Clinical Practice guidelines and comply with UK legislation and Warwick standard operating procedures (SOPs). All data will be stored securely and held in accordance with applicable data protection legislation.

## Consolidated Standards of Reporting Trials

The trial will be reported in line with the Consolidated Standards of Reporting Trials (CONSORT) statement using the non-pharmacological treatment interventions extension.

## Aim

Our aim is to determine which of two immobilisation strategies for people with a fractured ankle is superior.

## Objectives

### Primary objective

To quantify and draw inferences on observed differences in the Olerud and Molander Ankle Score (OMAS) between functional brace and plaster cast treatment groups 16 weeks after randomisation.

**Secondary objectives**

1. To quantify and draw inferences on observed differences in ankle function assessed using the OMAS score at 6 weeks, 10 weeks, 24 weeks and 24 months and Manchester-Oxford Foot Questionnaire (MOXFQ) 16 weeks after randomisation.
2. To draw inferences on the observed differences in ankle function assessed using the OMAS scores in operative and non-operative subgroups.
3. To draw inferences on the observed differences in ankle function assessed using the OMAS scores in those 50 years of age and over and those 49 years of age and under subgroups.
4. To quantify and draw inferences on observed differences in health-related quality of life (EuroQOL-five dimensions five-level, EQ-5D-5) between trial treatment groups at 6 weeks, 10 weeks, 16 weeks, 24 weeks, 12 months, 18 months and 24 months after randomisation.
5. To quantify and draw inferences on observed differences in Disability Rating Index (DRI) between trial treatment groups at 6 weeks, 10 weeks, 16 weeks, 24 weeks and 24 months after randomisation.
6. To quantify and draw inferences on observed differences on complication rates between trial treatment groups at 6 weeks, 10 weeks, 16 weeks, 24 weeks and 24 months after randomisation.
7. To estimate comparative cost–utility of the two trial treatment groups and collect resource use data at 6 weeks, 10 weeks, 16 weeks, 24 weeks, 12 months, 18 months and 24 months after randomisation.

## Methods and analysis

### Study design

This is a UK multicentre two arm parallel group randomised controlled trial.

### Sample size

The primary outcome for this study is the OMAS 16 weeks postinjury. The OMAS is measured on a scale between 0 and 100, where higher scores denote better function. We are seeking to show a between-group difference of 10 points. This is consistent with the Ankle Injury Management (AIM) study,^11^ which set the OMAS equivalence margin between groups to be 6 points. It is also consistent with other similar outcome measures such as the Foot and Ankle Outcome Score,^12^ and Visual Analogue Pain Scores in acute injury; that set Minimally Clinically Important Difference (MCIDs) of approximately 10 points on a 100 point scale in a trial context.

The SD of the OMAS from previous feasibility work was approximately 28 points. To account for any variation arising from recruiting from multiple study centres, we have selected a conservative estimation of the trial SD of 30 points. The total trial sample size required to detect a difference of 10 points given an SD of 30 points with two-sided significance set at 5% and 90% power is 382 participants. Allowing a margin of 20% loss during follow-up, this gives a minimum figure of 478 participants. If possible, recruiting a larger sample would enable the between-group differences of the two groups to be estimated with higher precision. The minimum 382 participants would create a 95% CI of width 8.5 points. If, for example, 625 participants were recruited, this would yield data on around 500 participants at 20% lost to follow-up and would enable a 95% CI of width 7.4 points to be constructed. Given that both interventions are routinely used in clinical practice and are low risk, if recruitment rates are higher than expected then recruitment will continue to a predefined end recruitment date to allow greater precision.

### Outcome measures

#### Primary

OMAS is a self-administered questionnaire consisting of nine different items: pain, stiffness, swelling, stair climbing, running, jumping, squatting, supports and work/activities of daily living.^13^ These data will be collected at 6 weeks, 10 weeks, 16 weeks, 24 weeks and 24 months after randomisation.

#### Secondary

EQ-5D-5L is a validated, generic health-related quality of life measure consisting of five dimensions each with a five-level response. Each combination of answers is converted into a health utility score that has good test–retest reliability, is simple for participants to use, and gives a single preference-based index value for health status that can be used for broader cost-effectiveness comparative purposes.^14^ These data will be collected at 6 weeks, 10 weeks, 16 weeks, 24 weeks, 12, 18 and 24 months after randomisation.

MOXFQ is a self-administered questionnaire that consists of 16 items, each with five response options comprising three separate underlying dimensions; walking/standing problems (seven items), foot pain (five items) and issues related to social interaction (four items). Item responses are each scored from 0 to 4, with 4 representing the most severe state. The scale scores representing each dimension are calculated by summing the responses to each item within that dimension. Raw scale scores are then converted to a metric (0–100; 100=most severe). These data will be collected at 16 weeks only.

DRI is a self-administered questionnaire. It consists of 12 items specifically related to function of the lower limb. The DRI has been proven to be a practical clinical and research instrument, with good responsiveness and acceptability for assessment of disability caused by impairment in the lower limb.^15^ These data will be collected at 6 weeks, 10 weeks, 16 weeks, 24 weeks and 24 months after randomisation.

Complications will be captured through two mechanisms. First, participants will be asked on each follow-up questionnaire collected at 6 weeks, 10 weeks, 16 weeks, 24 weeks, 12, 18 and 24 months after randomisation. Second, sites will be asked to report complications during the follow-up period to the central trial team. All prereduction baseline X-ray/radiographs and also the last X-ray/radiograph taken before the primary outcome point of 16 weeks will be collected as part of the complication data set.

The primary analysis will concentrate on direct intervention and healthcare/personal social services costs, while wider impact (societal) costs will be included within the sensitivity analyses. Relevant resource use questionnaires will be administered to participants at baseline and all follow-up points, to collect resource use data associated with the interventions under examination. These data will be collected at 6 weeks, 10 weeks, 16 weeks, 24 weeks, 12, 18 and 24 months after randomisation.

### Screening and eligibility

UK NHS trust sites will be used to screen all adults with a closed ankle fracture (within 3 weeks of operative management or injury if non-operative) which the treating clinician would consider plaster cast a reasonable management option will be screened by clinical care. Screening logs will be collected to assess the main reasons for exclusion as well as number of people unwilling to take part.

#### Inclusion criteria

1. Able to give written informed consent.
2. Aged 18 years or over.
3. A closed ankle fracture for which the treating clinician would consider plaster cast a reasonable management option.
4. Randomised within 3 weeks of operative management or injury if non-operative.

#### Exclusion criteria

1. Ankle fracture secondary to known metastatic disease.
2. Complex intra-articular fracture (eg, pilon fracture).
3. In the opinion of the surgeon the patient would require manipulation and close contact/moulded casting.
4. Wound complications contraindicating functional brace intervention.
5. Known pre-existing neuropathic joint disease contraindicating functional brace intervention.
6. Previous ankle fracture already randomised in the present trial.
7. Unable to adhere to trial procedures or complete postal questionnaires.

### Consent

Potential participants will be provided with verbal and written information about the study. Written informed consent will be obtained by a member of the research team at each site. The right of a potential participant to refuse participation without giving reasons will be respected, and recorded on the screening log. The participant will remain free to withdraw at any time without giving reasons and without prejudice to any further treatment, and will be provided with a contact point where he/she may obtain further information about the trial.

At the point of initial consent, participants will be asked if they consent to be contacted for any future research related to their ankle fracture. Those who consent will be provided with additional verbal and written information about appropriate additional studies related to their ankle fracture.

### Randomisation

Subjects will be randomised in ratio of 1:1 to the two study intervention arms strictly sequentially, as they become registered as eligible for randomisation on a secure password-protected web-based system. Allocation concealment will be maintained by an independent randomisation team, at the accredited clinical trials unit (CTU), who will be responsible for generation of the sequence and will have no role in the allocation of participants.

The treatment group will be allocated by computer using a minimisation algorithm with a random element and stratification by centre, age and operative/non-operative management following use of a secure web-based randomisation service. The randomisation system will allocate each participant a unique trial number.

Stratification by centre will help ensure that any clustering effect related to the centre itself will be equally distributed in the trial arms. Stratification on the basis of age will be used to ensure that younger participants with normal bone quality sustaining high-energy fractures and older participants with low-energy (fragility) fractures related to osteoporosis are balanced between arms. The age group stratification will be used as a proxy for bone density. Based on previous literature, this will be set at those 49 years and under and those 50 years and over.^1 16^ A final stratification based on operative/non-operative presentation will be implemented.

### Postrandomisation withdrawals

Unless a participant explicitly withdraws their consent, they will be followed up and data collected as per the protocol until the end of the trial. For participants explicitly withdrawing consent for follow-up procedures, trial data obtained up until the point of withdrawal will be included in the final analysis of the study. Participants will have the option to withdraw from the trial-related questionnaires, but continue to provide routine NHS data for the purposes of the trial, for example, hospital records of subsequent treatment for the ankle fracture. Participants who withdraw will not be replaced. Participants may also be withdrawn from the trial at the discretion of the investigator and/or trial steering committee (TSC) due to safety concerns.

### Interventions

All participants who require ankle fixation will have this performed according to the preferred technique of the operating surgeon, details of the procedure undertaken will be recorded. All participants will then receive normal local care until satisfactory clinical wound check (usually 2 weeks postoperatively, up to maximum 3 weeks), at which point the intervention will be applied by a member of the trauma team.

All participants not undergoing surgery will be approached to take part in the trial on first presentation to the trauma team fracture clinic, and will be eligible for trial participation up to a maximum of 3 weeks from injury. Weight-bearing status will be at the discretion of the treating member of the trauma team and recorded on subsequent case report forms (CRFs).

#### Control group: standard plaster cast

Participants will be fitted with cast immobilisation for a minimum of 3 weeks. It is expected that the control intervention will not exceed 8 weeks, however, this will be recorded and monitored by the trial management group (TMG). The details of the plaster cast material and method of application will be at the discretion of the treating clinician as per local procedures. Once the cast is removed, participants will be encouraged to complete active unloaded ankle range of movement exercises, as per routine clinical practice.

#### Intervention group: functional bracing

Participants will be fitted with a functional brace (A fixed angle design was specified but the brand and manufacturer was at the discretion of the local site) for a minimum of 3 weeks. It is expected that the intervention will not exceed 8 weeks, however, this will be recorded and monitored by the TMG. Throughout this period participants will be encouraged to remove their functional brace to complete active unloaded ankle range of movement exercises, little and often as pain allows. An information sheet explaining these exercises will be handed out to all participants randomised to this trial arm.

### Rehabilitation

Any other rehabilitation input will be left to the discretion of the treating trauma team member. However, a record of any additional rehabilitation, together with a record of any other interventions will be recorded on follow-up CRFs.

### Blinding

The participants cannot be blind to their treatment. The treating clinical team cannot be blind either, but will take no part in the assessment of participants.

All questionnaire data will be collected via phone and postal mechanisms and entered onto the trial central database by a member of the research team, who cannot be blinded to the trial interventions. Data will be presented to the data monitoring committee (DMC) using a combination of open and closed reports that will be further detailed in the statistical analysis plan.

### Adverse event management

Adverse events will be listed on CRF for return to the ‘AIR’ central office. Serious adverse events (SAEs) will be entered onto the SAE reporting form and reported to the central study team. All participants experiencing SAEs will be followed up as per protocol until the end of the trial. All SAEs that occur between date of consent and 24-month follow-up point will be reported to the sponsor (University of Warwick), ethics committee and oversight committees.

### Patient and public involvement

Patients were consulted to ascertain if the research gaps highlighted by peer-reviewed literature were of importance from their perspective. Based on these responses a single site feasibility trial was developed and funded by National Institute for Health Research (NIHR) (ISRCTN17809322). The feasibility trial team involved two patient and public involvement representatives, who continued their roles through to planning, development and delivery of the current main trial. Specifically, they have been active members on the TMG, reviewed consent procedures and developed patient facing study materials. At the end of the study, they will be vital to dissemination plans, which include the development of a lay summary of findings to be distributed through a variety of resources such as the trial website and posted directly to trial participants.

### End of trial

The trial will end when all participants have completed their 24-month follow-up. The trial will be stopped prematurely if mandated by the ethics committee, following recommendations from relevant oversight committees or funding for the trial ceases. The research ethics committee will be notified in writing within 90 days when the trial has been concluded or within 15 days if terminated early.

### Trial oversight

#### Trial management group

The TMG, consisting of the project staff and coinvestigators involved in the day-to-day running of the trial, will meet monthly throughout the project. Significant issues will be referred to the TSC or Investigators, as appropriate.

#### Trial steering committee

The TSC will have an independent chairperson and consist of clinicians, methodologists and lay representation. Meetings will be held not less than once a year. The TSC will take responsibility for approval of the protocol, major decisions (eg, need to change the protocol)l, monitoring progress of the trial, reviewing relevant information from other sources, considering recommendations from the DMC, informing and advising on all aspects of the trial.

#### Data monitoring committee

The DMC will consist of independent members with relevant clinical research and statistical expertise. The DMC meeting frequency will be guided by the DMC chair, but with a plan to be 6 months into the recruitment phase and regularly thereafter. Confidential reports containing recruitment, protocol compliance, safety data and interim assessments of outcomes will be reviewed by the DMC. The DMC will advise the TSC as to whether there is evidence or reason why any trial procedures should be modified or the study terminated.

#### Quality control

Quality assurance checks to ensure integrity of randomisation, study entry procedures and data collection will follow Warwick CTU SOPs.

### Statistical analysis plan

Treatment effects will be presented, with appropriate 95% CIs, for both the unadjusted and adjusted analyses. Tests will be two sided and considered to provide evidence for a significant difference if p values are less than 0.05 (5% significance level). All analyses will be conducted as intention to treat unless otherwise specified. No interim analyses are planned and interim analyses will be performed only where directed by the DMC, and with the agreement of the TSC.

Baseline data will be summarised to check comparability between treatment arms, and screening data will be checked to highlight any characteristic differences between those individuals in the study, and those eligible but withholding consent. A CONSORT chart illustrating participant flow throughout the study will also be produced. Standard statistical summaries will be presented for the primary outcome measure (OMAS) and all secondary outcome measures.

The main analysis will investigate differences in the primary outcome measure, 16 weeks after randomisation, between the two treatment groups. Unadjusted and adjusted regression analyses will be used to estimate the between-group difference. The adjusted analyses will adjust for the stratification variables, baseline scores and any other clinically important variables. More specifically, adjusted mixed-effects modelling will be used where the recruiting centre will be included as a random effect to allow for possible heterogeneity in patient outcomes due to the recruiting centre. Since individual clinicians will treat only a small number of participants enrolled in the trial, we do not expect clinician-specific effects to be important in this study and hence will not be modelled. This adjusted mixed-effects linear regression analysis will be reported as the primary analysis, and will be used to assess evidence for differences in outcomes between intervention arms.

Descriptive statistics of patient-reported outcome measure (PROM) data (ie, OMAS, MOXFQ, EQ5D and DRI) at each time point will be calculated with between-group analyses following the method set out for the primary analysis above. Patterns of recovery will also be explored. For example, the area under the curve for PROM data will be estimated using the trapezoidal rule for each allocation arm.

Complications will be summarised with between-groups comparisons evaluated using X^2^ tests. Temporal patterns of any complications will be presented graphically and if appropriate, a time-to-event analysis (eg, Kaplan-Meier survival analysis) will be used to assess the overall risk and risk within individual classes of important complications (eg, non-union).

Two prespecified subgroup analyses will be undertaken to assess whether there is evidence that the intervention effect differs between whether the study participants receives operative or non-operative treatment prior to the study intervention and study participants are aged 50 or over at study randomisation.

The subgroup analyses will follow the methods described for the primary analysis, with additional interaction terms incorporated into the mixed-effects regression model to assess the level of support for these hypotheses. The study is not powered to formally test these hypotheses, so they will be reported as exploratory analyses only, and as subsidiary to the analysis reporting the main effects of the intervention in the full study population.

Some data may not be available due to voluntary withdrawal of participants, lack of completion of individual data items or general lost to follow-up. Where possible the reasons for data ‘missingness’ will be ascertained and reported. The nature and pattern of the missingness will be carefully considered, including whether data can be treated as missing completely at random. If judged appropriate, missing data will be imputed using the multiple imputation facilities available in the statistical analysis software.

If imputation is undertaken, the resulting imputed datasets will be analysed, together with appropriate sensitivity analyses. Any imputation methods used for scores and other derived variables will be carefully considered and justified. Reasons for ineligibility, non-compliance, withdrawal or other protocol violations will be stated and any patterns summarised. More formal analysis, for example, using logistic regression with ‘protocol violation’ as a response, may also be appropriate and aid interpretation.

### Health economic analysis plan

Prospective economic evaluation, conducted from an NHS and personal social services perspective, will be included. The economic evaluation will estimate the difference in the cost of resource inputs between the two intervention groups, enabling costs and consequences to be compared. The methods will adhere to the recommendations of the National Institute for Health and Care Excellence (NICE) reference case.^17^

Primary research methods will be followed to estimate the costs of the treatment options, including resource inputs associated with the plaster materials and braces, supplementary interventions, adverse events and rehabilitation inputs. Broader resource utilisation associated with the ankle injury will be captured through routine health service data collection systems and participant questionnaires administered at each follow-up time point.

Unit costs will be estimated from local and national sources in addition to primary research using established accounting methods. Costs will be standardised to current prices where possible. Health-related quality of life will be measured at the time of consent, and all follow time points using the EQ-5D-5L measure. Responses will be used to generate quality-adjusted life years (QALYs) using the UK time-trade-off value set recommended by the EQ group.^18^

Within-trial analysis using bivariate regression of costs and QALYs, with multiple imputation of missing data, will inform a probabilistic assessment of incremental treatment cost-effectiveness from a health service perspective. Missingness mechanisms will be explored and multiple imputation methods will be used where appropriate to avoid biases associated with complete case analysis. Costs and outcomes arising after the first year of the trial will be discounted at 3.5%. Sensitivity analyses will be undertaken to explore uncertainty on the incremental cost-effectiveness ratios and to consider issues of generalisability of the study.

More extensive economic modelling using decision-analytical methods may be considered to extend the target population, time horizon and decision context, drawing on the best available information from the literature and stakeholder consultations to supplement the trial data. Parameter uncertainty in the decision-analytical model will be explored using probabilistic sensitivity analysis. Longer term costs and consequences will be discounted to present values using discount rates recommended for health technology appraisal in the UK (current discount rate: 3.5%).

### Ethics and dissemination

Both functional brace and plaster interventions are currently used across the NHS for the management of ankle fractures. Consequently, both trial interventions reflect current standard practice and do not expose trial participants to any substantial risks over and above standard care currently received.

The results of the trial will be reported first to trial collaborators. The main report will be drafted by the trial coordinating team, and the final version will be agreed by the TSC before submission for publication, on behalf of the collaboration. The trial will be reported in accordance with the CONSORT (http://www.consort-statement.org).

The results of this project, full protocol and related documentation will be disseminated through the trial website, peer-reviewed journals, conference presentations among the orthopaedic and rehabilitation networks, the National Library for Health, policy-makers such as NICE, patient-specific newsletters and through local mechanisms at all participating centres.

## Supplementary Material

Reviewer comments

Author's manuscript
