# Supplementary material for: Cast versus functional brace in the rehabilitation of patients treated for an ankle fracture: protocol for the UK study of ankle injury rehabilitation (AIR) multicentre randomised trial
Source: BMJ Open. 2018 Dec 18;8(12):e027242. doi: 10.1136/bmjopen-2018-027242 (PMC6303686; doi:10.1136/bmjopen-2018-027242)
Supplement: Author's manuscript [file bmjopen-2018-027242.draft_revisions.pdf]

BMJ Open is committed to open peer review. As part of this commitment we make the peer review history of every article we publish publicly available.

When an article is published we post the peer reviewers' comments and the authors' responses online. We also post the versions of the paper that were used during peer review. These are the versions that the peer review comments apply to.

The versions of the paper that follow are the versions that were submitted during the peer review process. They are not the versions of record or the final published versions. They should not be cited or distributed as the published version of this manuscript.

BMJ Open is an open access journal and the full, final, typeset and author-corrected version of record of the manuscript is available on our site with no access controls, subscription charges or pay-per-view fees (http://bmjopen.bmj.com).

If you have any questions on BMJ Open's open peer review process please email editorial.bmjopen@bmj.com

BMJ Open

Cast versus functional brace in the rehabilitation of patients treated for an ankle fracture: Protocol for the UK study of ankle injury rehabilitation (AIR) multi-centre randomised trial

Journal:	*BMJ Open*
Manuscript ID	bmjopen-2018-027242
Article Type:	Protocol
Date Submitted by the Author:	15-Oct-2018
Complete List of Authors:	Kearney, Rebecca; University of Warwick, Warwick Medical School<br>McKeown, Rebecca; University of Warwick, Warwick Medical School<br>Stevens, Siobhan; University of Warwick, Warwick Medical School<br>Parsons, Nicholas; University of Warwick, Warwick Medical School<br>Parsons, Helen; University of Warwick, Warwick Medical School<br>Wells, Philip; University of Warwick, Warwick Medical School<br>Brown, Jaclyn; University of Warwick, Warwick Medical School<br>Underwood, Martin; University of Warwick, Warwick Medical School<br>Redmond, Anthony; University of Leeds, Leeds Institute of Rheumatology and Musculoskeletal Medicine<br>Mason, James; University of Warwick, Warwick Medical School<br>Costa, Matthew; University of Oxford, Nuffield Department of Orthopaedics, Rheumatology, and Musculoskeletal Sciences
Keywords:	Rehabilitation medicine < INTERNAL MEDICINE, Foot & ankle < ORTHOPAEDIC & TRAUMA SURGERY, Adult orthopaedics < ORTHOPAEDIC & TRAUMA SURGERY

SCHOLARONE™
Manuscripts

TITLE PAGE

Title

Cast versus functional brace in the rehabilitation of patients treated for an ankle fracture: Protocol for the UK study of ankle injury rehabilitation (AIR) multi-centre randomised trial

Corresponding Author

Rebecca S Kearney: Associate Professor, University of Warwick, Warwick Medical School, Gibbet Hill Road, Coventry, CV4 7AL; R.S.Kearney@warwick.ac.uk; 02476 573156

Co-authors

- Rebecca McKeown: Trial Manager, University of Warwick, Warwick Medical School, Gibbet Hill Road, Coventry, CV4 7AL (R.McKeown.1@warwick.ac.uk)
- Siobhan Stevens: Trial Manager, University of Warwick, Warwick Medical School, Gibbet Hill Road, Coventry, CV4 7AL (Siobhan.Stevens@warwick.ac.uk)
- Nick Parsons: Associate Professor in Medical Statistics, University of Warwick, Warwick Medical School, Gibbet Hill Road, Coventry, CV4 7AL (Nick.Parsons@warwick.ac.uk)
- Helen Parsons: Senior Research Fellow, University of Warwick, Warwick Medical School, Gibbet Hill Road, Coventry, CV4 7AL (H.Parsons@warwick.ac.uk)
- Philip Wells: Research Associate, University of Warwick, Warwick Medical School, Gibbet Hill Road, Coventry, CV4 7AL (Philip.Wells@warwick.ac.uk)
- Jaclyn Brown: Senior Project Manager, University of Warwick, Warwick Medical School, Gibbet Hill Road, Coventry, CV4 7AL (J.Brown.10.@warwick.ac.uk)
- Martin Underwood: Professor, University of Warwick, Warwick Medical School, Gibbet Hill Road, Coventry, CV4 7AL (M.Underwood@warwick.ac.uk)
- Anthony C Redmond: Professor of Clinical Biomechanics, University of Leeds, Leeds Institute for Rheumatic and Musculoskeletal Medicine, Chapel Allerton Hospital, Chapeltown Road, Leeds, LS7 4SA (A.Redmond@leeds.ac.uk)
- Matthew Costa: Professor of Orthopaedic Trauma, Oxford University, Kadoorie Centre, Level 3, John Radcliffe Hospital, Oxford, OX3 9DU, UK (matthew.costa@ndorms.ox.ac.uk)
- James Mason: Professor, University of Warwick, Warwick Medical School, Gibbet Hill Road, Coventry, CV4 7AL (J.Mason@warwick.ac.uk)

For peer review only

For peer review only - http://bmjopen.bmj.com/site/about/guidelines.xhtml

Keywords: Ankle, fracture, rehabilitation, immobilisation

Word Count: 4,189

ABSTRACT

Introduction

Each year in the UK over 120,000 people fracture their ankle. It is not known what the best rehabilitation strategy is for these people. Traditionally standard care has involved immobilisation in a plaster cast but an alternative is a functional brace, which can be removed to allow early movement. This paper details the protocol for a multi-centre randomised trial of plaster cast immobilisation versus functional bracing for patients with an ankle fracture.

Methods and analysis

We will recruit adults with a fractured ankle, for which the treating clinician would consider plaster cast to be a reasonable management option. Randomisation will be on a 1:1 basis, stratified by centre, operative or non-operative management and age. Participants will be allocated to either plaster cast or a functional brace, both treatments are widely used. To have 90% power to detect a difference of 10 points on the primary outcome (Olerud and Molander Ankle Score) at the primary outcome time point (16 weeks) we need to randomise a minimum of 478 people. Quality of life and resource use will be collected at six, 10, 16, 24 weeks and 12, 18, 24 months. The differences between treatment groups will be assessed on an intention-to-treat basis. The economic evaluation will adhere to the recommendations of the NICE Reference Case.

Ethics, registration and dissemination

This trial is funded by the National Institute for Health Research commencing 1st January 2017 (NIHR; CDF-2016-09-009). National Research Ethic Committee approved this study on 4th July 2017 (17/WM/0239). It was registered on the International Standard Randomised Controlled Trial Number registry with reference no ISRCTN15537280 on 24th July 2017. The first site opened to recruitment 9th October 2017. The results of this trial will be submitted to a peer-reviewed journal and will inform clinical practice.

ARTICLE SUMMARY: STRENGHTS AND LIMITATIONS OF THIS STUDY

- Broad eligibility criteria to ensure results can be generalised to the wider population.
- UK wide trial across a minimum of 19 centres to optimize external validity

For peer review only - http://bmjopen.bmj.com/site/about/guidelines.xhtml

- Primary outcome measure is patient centred.
- Blinding of interventions is not possible.

MAIN TEXT

Introduction

Nine percent of trauma surgeons' workload in the UK is managing ankle fractures; over 120,000 per year in the UK. A threefold increase is expected by 2030 due to an increase in older adults who are remaining physically active (1, 2). The frequency of this injury is an increasing burden on the NHS year on year (3). The short-term impact of this injury results in physical impairments of pain, stiffness, weakness and swelling. The longer-term impact results in extended time off work and development of posttraumatic arthritis (4).

Management has traditionally included plaster cast immobilisation for several weeks, whilst the bone heals. A cast provides maximum support, however there are potential problems, there are the risks associated with prolonged immobilisation such as muscle atrophy, deep vein thrombosis and joint stiffness. There are the long-term consequences, which include prolonged gait abnormalities, persistent calf muscle weakness and an inability to return to previous activity levels. Functional bracing may address these issues (5). However, it does not provide the same degree of support to the healing bones.

A research priority exercise was undertaken by a UK orthopaedic group in 2010 (6). One of the top priority questions was to establish whether there is a functional advantage associated with different rehabilitation plans following an ankle fracture. This was followed by a 2012 Cochrane review (7) which identified the need for further research into the optimal method of immobilisation for both operatively and non-operatively managed acute ankle fractures. This issue was raised again in a James Lind Alliance Priority Setting Partnership in 2018, which included: '*What is the best physiotherapy regime for adults during out-of-hospital recovery from a fragility fracture of the lower limb?*' in its top ten priority research questions (8).

Good Clinical Practice

The trial will be conducted in full conformance with the principles of the Declaration of Helsinki and to Good Clinical Practice guidelines and comply with UK legislation and Warwick Standard Operating

For peer review only - http://bmjopen.bmj.com/site/about/guidelines.xhtml

Procedures (SOPs). All data will be stored securely and held in accordance with applicable data protection legislation.

CONSORT

The trial will be reported in line with the CONSORT statement using the non-pharmacological treatment interventions extension.

Aim

Our aim is to determine which of two immobilisation strategies for people with a fractured ankle is superior.

Objectives

Primary Objective

To quantify and draw inferences on observed differences in the Olerud and Molander Ankle Score (OMAS) between functional brace and plaster cast treatment groups sixteen weeks after randomisation.

Secondary Objectives

1) To quantify and draw inferences on observed differences in ankle function assessed using the OMAS score at six weeks, 10 weeks, 24 weeks and 24 months and Manchester-Oxford Foot Questionnaire (MOXFQ) 16 weeks after randomisation.

2) To draw inferences on the observed differences in ankle function assessed using the OMAS scores in operative and non-operative subgroups

3) To draw inferences on the observed differences in ankle function assessed using the OMAS scores in those 50 years of age and over and those 49 years of age and under subgroups

4) To quantify and draw inferences on observed differences in health related quality of life (EQ5D-5) between trial treatment groups at six weeks, 10 weeks, 16 weeks, 24 weeks, 12 months, 18 months and 24 months after randomisation.

5) To quantify and draw inferences on observed differences in Disability Rating Index (DRI) between trial treatment groups at six weeks, 10 weeks, 16 weeks, 24 weeks and 24 months after randomisation.

6) To quantify and draw inferences on observed differences on complication rates between trial treatment groups at six weeks, 10 weeks, 16 weeks, 24 weeks and 24 months after randomisation.

For peer review only - http://bmjopen.bmj.com/site/about/guidelines.xhtml

7) To estimate comparative cost-utility of the two trial treatment groups and collect resource use data at six weeks, 10 weeks, 16 weeks, 24 weeks, 12 months, 18 months and 24 months after randomisation.

Methods and analysis

Study design

This is a UK multi-centre two arm parallel group randomised controlled trial.

Sample size

The primary outcome for this study is the OMAS 16 weeks post injury. The OMAS is measured on a scale between 0 and 100, where higher scores denote better function. We are seeking to show a between group difference of 10 points. This is consistent with the AIM study (9), which set the OMAS equivalence margin between groups to be 6 points. It is also consistent with other similar outcome measures such as the Foot and Ankle Outcome Score (10), and visual analogue pain scores in acute injury; that set MCIDs of approximately 10 points on a 100 point scale in a trial context.

The standard deviation (SD) of the OMAS from previous feasibility work was approximately 28 points. To account for any variation arising from recruiting from multiple study centres, we have selected a conservative estimation of the trial SD of 30 points. The total trial sample size required to detect a difference of 10 points given a SD of 30 points with two-sided significance set at 5% and 90% power is 382 participants. Allowing a margin of 20% loss during follow-up, this gives a minimum figure of 478 participants. If possible, recruiting a larger sample would enable the between group differences of the two groups to be estimated with higher precision. The minimum 382 participants would create a 95% CI of width 8.5 points. If, for example, 625 participants were recruited, this would yield data on around 500 participants at 20% loss to follow up and would enable a 95% confidence interval (CI) of width 7.4 points to be constructed. Given that both interventions are routinely used in clinical practice and are low risk, if recruitment rates are higher than expected then recruitment will continue to a pre-defined end recruitment date to allow greater precision.

Outcome measures

Primary

Olerud and Molander Ankle Score (OMAS) is a self-administered questionnaire consisting of nine different items; pain, stiffness, swelling, stair climbing, running, jumping, squatting, supports and

For peer review only - http://bmjopen.bmj.com/site/about/guidelines.xhtml

work/activities of daily living (11). These data will be collected at six weeks, 10 weeks, 16 weeks, 24 weeks and 24 months after randomisation.

Secondary

EQ-5D-5L is a validated, generic health-related quality of life measure consisting of five dimensions each with a 5-level response. Each combination of answers is converted into a health utility score that has good test-retest reliability, is simple for participants to use, and gives a single preference-based index value for health status that can be used for broader cost-effectiveness comparative purposes (12). These data will be collected at six weeks, 10 weeks, 16 weeks, 24 weeks, 12, 18 and 24 months after randomisation.

MOXFQ is a self-administered questionnaire that consists of 16 items, each with 5 response options comprising 3 separate underlying dimensions; Walking/standing problems (7 items), foot pain (5 items) and issues related to social interaction (4 items). Item responses are each scored from 0 to 4, with 4 representing the most severe state. The scale scores representing each dimension are calculated by summing the responses to each item within that dimension. Raw scale scores are then converted to a metric (0-100; 100=most severe). These data will be collected at 16 weeks only.

DRI is a self-administered questionnaire. It consists of 12 items specifically related to function of the lower limb. The DRI has been proven to be a practical clinical and research instrument, with good responsiveness and acceptability for assessment of disability caused by impairment in the lower limb (13). These data will be collected at 6 weeks, 10 weeks, 16 weeks, 24 weeks and 24 months after randomisation.

Complications will be captured through two mechanisms. Firstly Participants will be asked on each follow up questionnaire collected at 6 weeks, 10 weeks, 16 weeks, 24 weeks, 12, 18 and 24 months after randomisation. Secondly sites will be asked to report complications during the follow up period to the central trial team. All pre-reduction baseline X-Ray/radiographs and also the last X-Ray/radiograph taken before the primary outcome point of 16 weeks will be collected as part of the complication data set.

The primary analysis will concentrate on direct intervention and healthcare/personal social services costs, while wider impact (societal) costs will be included within the sensitivity analyses. Relevant

For peer review only - http://bmjopen.bmj.com/site/about/guidelines.xhtml

resource use questionnaires will be administered to participants at baseline and all follow-up points, to collect resource use data associated with the interventions under examination. These data will be collected at six weeks, 10 weeks, 16 weeks, 24 weeks, 12, 18 and 24 months after randomisation.

Screening and Eligibility

UK NHS trust sites will be used to screen all adults with a closed ankle fracture (within three weeks of operative management or injury if non-operative) which the treating clinician would consider plaster cast a reasonable management option will be screened by clinical care. Screening logs will be collected to assess the main reasons for exclusion as well as number of people unwilling to take part.

Inclusion criteria

1. Able to give written informed consent
2. Aged 18 years or over
3. A closed ankle fracture for which the treating clinician would consider plaster cast a reasonable management option
4. Randomised within three weeks of operative management or injury if non-operative

Exclusion criteria

1. Ankle fracture secondary to known metastatic disease
2. Complex intra-articular fracture (e.g. Pilon fracture)
3. In the opinion of the surgeon the patient would require manipulation and close contact/moulded casting
4. Wound complications contraindicating functional brace intervention
5. Known pre-existing neuropathic joint disease contraindicating functional brace intervention
6. Previous ankle fracture already randomised in the present trial
7. Unable to adhere to trial procedures or complete postal questionnaires

Consent

Potential participants will be provided with verbal and written information about the study. Written informed consent will be obtained by a member of the research team at each site. The right of a potential participant to refuse participation without giving reasons will be respected, and recorded on the screening log. The participant will remain free to withdraw at any time without giving reasons and

For peer review only

without prejudice to any further treatment, and will be provided with a contact point where he/she may obtain further information about the trial.

At the point of initial consent participants will be asked if they consent to be contacted for any future research related to their ankle fracture. Those who consent will be provided with additional verbal and written information about appropriate additional studies related to their ankle fracture.

Randomisation

Subjects will be randomised in ratio of 1:1 to the two study intervention arms strictly sequentially, as they become registered as eligible for randomisation on a secure password protected web based system. Allocation concealment will be maintained by an independent randomisation team, at the accredited Clinical Trials Unit, who will be responsible for generation of the sequence and will have no role in the allocation of participants.

The treatment group will be allocated by computer using a minimisation algorithm with a random element and stratification by centre, age and operative/non operative management following use of a secure web based randomisation service. The randomisation system will allocate each participant a unique trial number.

Stratification by centre will help ensure that any clustering effect related to the centre itself will be equally distributed in the trial arms. Stratification on the basis of age will be used to ensure that younger participants with normal bone quality sustaining high-energy fractures and older participants with low-energy (fragility) fractures related to osteoporosis are balanced between arms. The age group stratification will be used as a proxy for bone density. Based on previous literature this will be set at those 49 years and under and those 50 years and over (1, 14). A final stratification based on operative/non operative presentation will be implemented.

Post randomisation withdrawals

Unless a participant explicitly withdraws their consent, they will be followed-up and data collected as per the protocol until the end of the trial. For participants explicitly withdrawing consent for follow up procedures, trial data obtained up until the point of withdrawal will be included in the final analysis of the study. Participants will have the option to withdraw from the trial-related questionnaires, but continue to provide routine NHS data for the purposes of the trial e.g. hospital records of subsequent

For peer review only - http://bmjopen.bmj.com/site/about/guidelines.xhtml

treatment for the ankle fracture. Participants who withdraw will not be replaced. Participants may also be withdrawn from the trial at the discretion of the investigator and/or Trial Steering Committee due to safety concerns.

Interventions

All participants who require ankle fixation will have this performed according to the preferred technique of the operating surgeon, details of the procedure undertaken will be recorded. All participants will then receive normal local care until satisfactory clinical wound check (usually two weeks post operatively, up to maximum three weeks), at which point the intervention will be applied by a member of the trauma team.

All participants not undergoing surgery will be approached to take part in the trial on first presentation to the trauma team fracture clinic, and will be eligible for trial participation up to a maximum of three weeks from injury. Weight bearing status will be at the discretion of the treating member of the trauma team and recorded on subsequent CRFs.

Control Group - Standard Plaster Cast

Participants will be fitted with cast immobilisation for a minimum of three weeks. It is expected that the control intervention will not exceed eight weeks, however this will be recorded and monitored by the Trial Management group (TMG). The details of the plaster cast material and method of application will be at the discretion of the treating clinician as per local procedures. Once the cast is removed participants will be encouraged to complete active unloaded ankle range of movement exercises, as per routine clinical practice.

Intervention Group - Functional Bracing

Participants will be fitted with a functional brace (A fixed angle design was specified but the brand and manufacturer was at the discretion of the local site) for a minimum of three weeks. It is expected that the intervention will not exceed eight weeks, however this will be recorded and monitored by the TMG. Throughout this period participants will be encouraged to remove their functional brace to complete active unloaded ankle range of movement exercises, little and often as pain allows. An information sheet explaining these exercises will be handed out to all participants randomised to this trial arm.

For peer review only - http://bmjopen.bmj.com/site/about/guidelines.xhtml

Rehabilitation

Any other rehabilitation input will be left to the discretion of the treating trauma team member. However, a record of any additional rehabilitation, together with a record of any other interventions will be recorded on follow up CRFs.

Blinding

The participants cannot be blind to their treatment. The treating clinical team cannot be blind either, but will take no part in the assessment of participants.

All questionnaire data will be collected via phone and postal mechanisms and entered onto the trial central database by a member of the research team, who cannot be blinded to the trial interventions. Data will be presented to the Data monitoring committee (DMC) using a combination of open and closed reports that will be further detailed in the statistical analysis plan (SAP).

Adverse event management

Adverse events will be listed on Case Report Form for return to the 'AIR' central office. Serious adverse events (SAEs) will be entered onto the SAE reporting form and reported to the central study team. All participants experiencing SAEs will be followed-up as per protocol until the end of the trial. All SAEs that occur between date of consent and 24 month follow-up point will be reported to the sponsor (University of Warwick), ethics committee and oversight committees.

Patient and Public Involvement PPI

Patients were consulted to ascertain if the research gaps highlighted by peer reviewed literature were of importance from their perspective. Based on these responses a single site feasibility trial was developed and funded by NIHR (ISRCTN17809322). The feasibility trial team involved two PPI representatives, who continued their roles through to planning, development and delivery of the current main trial. Specifically, they have been active members on the trial management group, reviewed consent procedures and developed patient facing study materials. At the end of the study they will be vital to dissemination plans, which include the development of a lay summary of findings to be distributed through a variety of resources such as the trial website and posted directly to trial participants.

End of trial

For peer review only - http://bmjopen.bmj.com/site/about/guidelines.xhtml

The trial will end when all participants have completed their 24-month follow-up. The trial will be stopped prematurely if mandated by the Ethics Committee, following recommendations from relevant oversight committees or funding for the trial ceases. The Research Ethics Committee will be notified in writing within 90 days when the trial has been concluded or within 15 days if terminated early.

Trial oversight

Trial Management Group (TMG)

The TMG, consisting of the project staff and co-investigators involved in the day-to-day running of the trial, will meet monthly throughout the project. Significant issues will be referred to the Trial Steering Committee (TSC) or Investigators, as appropriate.

Trial Steering Committee (TSC)

The TSC will have an independent chairperson and consist of clinicians, methodologists and lay representation. Meetings will be held not less than once a year. The TSC will take responsibility for approval of the protocol, major decisions (e.g. need to change the protocol)l, monitoring progress of the trial, reviewing relevant information from other sources, considering recommendations from the DMC, informing and advising on all aspects of the trial.

Data Monitoring Committee (DMC)

The DMC will consist of independent members with relevant clinical research and statistical expertise. The DMC meeting frquency will be guided by the DMC chair, but with a plan to be six months into the recruitment phase and regularly thereafter. Confidential reports containing recruitment, protocol compliance, safety data and interim assessments of outcomes will be reviewed by the DMC. The DMC will advise the TSC as to whether there is evidence or reason why any trial procedures should be modified or the study terminated.

Quality Control

Quality assurance checks to ensure integrity of randomisation, study entry procedures and data collection will follow Warwick CTU standard operating procedures.

Statistical analysis plan

For peer review only - http://bmjopen.bmj.com/site/about/guidelines.xhtml

Treatment effects will be presented, with appropriate 95% confidence intervals, for both the unadjusted and adjusted analyses. Tests will be two-sided and considered to provide evidence for a significant difference if p-values are less than 0.05 (5% significance level). All analyses will be conducted as intention to treat unless otherwise specified. No interim analyses are planned and interim analyses will be performed only where directed by the DMC, and with the agreement of the TSC.

Baseline data will be summarised to check comparability between treatment arms, and screening data will be checked to highlight any characteristic differences between those individuals in the study, and those eligible but withholding consent. A CONSORT chart illustrating participant flow throughout the study will also be produced. Standard statistical summaries will be presented for the primary outcome measure (OMAS) and all secondary outcome measures.

The main analysis will investigate differences in the primary outcome measure, sixteen weeks after randomisation, between the two treatment groups. Unadjusted and adjusted regression analyses will be used to estimate the between group difference. The adjusted analyses will adjust for the stratification variables, baseline scores and any other clinically important variables. More specifically, adjusted mixed-effects modelling will be used where the recruiting centre will be included as a random effect to allow for possible heterogeneity in patient outcomes due to the recruiting centre. Since individual clinicians will treat only a small number of participants enrolled in the trial, we do not expect clinician specific effects to be important in this study and hence will not be modelled. This adjusted mixed-effects linear regression analysis will be reported as the primary analysis, and will be used to assess evidence for differences in outcomes between intervention arms.

Descriptive statistics of patient reported outcome measure (PROM) data (i.e. OMAS, MOXFQ, EQ5D and DRI) at each time point will be calculated with between group analyses following the method set out for the primary analysis above. Patterns of recovery will also be explored. For example, the area-under-the-curve (AUC) for PROM data will be estimated using the trapezoidal rule for each allocation arm

Complications will be summarised with between groups comparisons evaluated using chi-squared tests. Temporal patterns of any complications will be presented graphically and if appropriate, a time-to-event analysis (e.g. Kaplan-Meier survival analysis) will be used to assess the overall risk and risk within individual classes of important complications (e.g. non-union).

For peer review only - http://bmjopen.bmj.com/site/about/guidelines.xhtml

Two pre-specified sub-group analyses will be undertaken to assess whether there is evidence that the intervention effect differs between whether the study participants receives operative or non-operative treatment prior to the study intervention and study participants are aged 50 or over at study randomisation.

The subgroup analyses will follow the methods described for the primary analysis, with additional interaction terms incorporated into the mixed-effects regression model to assess the level of support for these hypotheses. The study is not powered to formally test these hypotheses, so they will be reported as exploratory analyses only, and as subsidiary to the analysis reporting the main effects of the intervention in the full study population.

Some data may not be available due to voluntary withdrawal of participants, lack of completion of individual data items or general loss to follow-up. Where possible the reasons for data 'missingness' will be ascertained and reported. The nature and pattern of the missingness will be carefully considered, including whether data can be treated as missing completely at random. If judged appropriate, missing data will be imputed using the multiple imputation facilities available in the statistical analysis software.

If imputation is undertaken, the resulting imputed datasets will be analysed, together with appropriate sensitivity analyses. Any imputation methods used for scores and other derived variables will be carefully considered and justified. Reasons for ineligibility, non-compliance, withdrawal or other protocol violations will be stated and any patterns summarised. More formal analysis, for example using logistic regression with 'protocol violation' as a response, may also be appropriate and aid interpretation.

Health economic analysis plan

Prospective economic evaluation, conducted from a NHS and personal social services perspective, will be included. The economic evaluation will estimate the difference in the cost of resource inputs between the two intervention groups, enabling costs and consequences to be compared. The methods will adhere to the recommendations of the NICE Reference Case(15).

For peer review only

For peer review only - http://bmjopen.bmj.com/site/about/guidelines.xhtml

Primary research methods will be followed to estimate the costs of the treatment options, including resource inputs associated with the plaster materials and braces, supplementary interventions, adverse events and rehabilitation inputs. Broader resource utilisation associated with the ankle injury will be captured through routine health service data collection systems and participant questionnaires administered at each follow up time point.

Unit costs will be estimated from local and national sources in addition to primary research using established accounting methods. Costs will be standardised to current prices where possible. Health-related quality of life will be measured at the time of consent, and all follow time points using the EQ-5D-5L measure. Responses will be used to generate quality-adjusted life years (QALYs) using the UK time-trade-off (TTO) value set recommended by the EuroQol group (16).

Within-trial analysis using bivariate regression of costs and QALYs, with multiple imputation of missing data, will inform a probabilistic assessment of incremental treatment cost-effectiveness from a health service perspective. Missingness mechanisms will be explored and multiple imputation methods will be used where appropriate to avoid biases associated with complete case analysis. Costs and outcomes arising after the first year of the trial will be discounted at 3.5%. Sensitivity analyses will be undertaken to explore uncertainty on the incremental cost-effectiveness ratios and to consider issues of generalisability of the study.

More extensive economic modelling using decision-analytic methods may be considered to extend the target population, time horizon and decision context, drawing on best available information from the literature and stakeholder consultations to supplement the trial data. Parameter uncertainty in the decision-analytic model will be explored using probabilistic sensitivity analysis. Longer term costs and consequences will be discounted to present values using discount rates recommended for health technology appraisal in the UK (current discount rate: 3.5%).

Ethics and dissemination

Edgbaston West Midlands Research Ethics Committee approved this study on 4th July 2017 (REC Reference 17/WM/0239). It was registered on the International Standard Randomised Controlled Trial Number registry (ISRCTN15537280) on 24th July 2017. Both functional brace and plaster interventions are currently used across the NHS for the management of ankle fractures. Consequently, both trial

For peer review only - http://bmjopen.bmj.com/site/about/guidelines.xhtml

interventions reflect current standard practice and do not expose trial participants to any substantial risks over and above standard care currently received.

The results of the trial will be reported first to trial collaborators. The main report will be drafted by the trial coordinating team, and the final version will be agreed by the TSC before submission for publication, on behalf of the collaboration. The trial will be reported in accordance with the Consolidated Standards of Reporting Trials (CONSORT) guidelines (www.consort-statement.org).

The results of this project, full protocol and related documentation will be disseminated through the trial website, peer-reviewed journals, conference presentations amongst the orthopaedic and rehabilitation networks, the National Library for Health, policy makers such as NICE, patient specific newsletters and through local mechanisms at all participating centres.

For peer review only

For peer review only - http://bmjopen.bmj.com/site/about/guidelines.xhtml

Author contributions and Acknowledgements

RSK, MLC, MU and ACR wrote the background section and developed the research question. RSK, MLC, MU, ACR, RM, JB, RM and SS wrote the research methodology and management sections of the protocol. NP, HP and PW wrote the sample size and statistical analysis sections of the protocol. JM wrote the health economic evaluation section of the protocol. All authors reviewed and approved the final manuscript.

The research team would like to acknowledge Richard Grant and Karen Keates for their contribution throughout the feasibility and main trial as PPI Representatives.

Funding

This trial was funded by the National Institute for Health Research commencing 1st January 2017, as part of a personal Fellowship to RSK (NIHR; CDF-2016-09-009).

Data Statement

The data sharing plans for the current study will be updated via ISRCTN

Disclaimer

The funder has not been involved in the design of the study. The views expressed are those of the authors and not necessarily those of the NHS, the NIHR or the Department of Health.

Competing interests

RSK is a member of the UK NIHR HTA CET board, NIHR ICA Doctoral panel and NIHR RfPB board. MLC is a member of the UK NIHR HTA General Board. ACR is a member of the NIHR ICA Clinical lectureship/Senior Clinical Lectureship panel and holder of a NIHR Senior Investigator award. RSK,MLC, ACR, NP, HP, PW, have all been award current and previous NIHR research grants.

MU was Chair of the NICE accreditation advisory committee until March 2017 for which he received a fee. He is chief investigator or co-investigator on multiple previous and current research grants from the UK National Institute for Health Research, Arthritis Research UK and is a co-investigator on grants funded by Arthritis Australia and Australian NHMRC. He has received travel expenses for speaking at conferences from the professional organisations hosting the

For peer review only - http://bmjopen.bmj.com/site/about/guidelines.xhtml

conferences He is a director and shareholder of Clinvivo Ltd that provides electronic data collection for health services research. He is part of an academic partnership with Serco Ltd. He is an editor of the NIHR journal series for which he receives a fee.

Provenance and peer review

Not commissioned; peer reviewed for ethical and funding approval for NIHR Fellowship prior to submission.

References

1. Court-Brown CM, Caesar B. Epidemiology of adult fractures: A review. Injury. 2006;37(8):691-7.
2. Kannus P, Palvanen M, Niemi S, et al. Increasing number and incidence of low-trauma ankle fractures in elderly people: Finnish statistics during 1970-2000 and projections for the future. Bone. 2002;31(3):430-3.
3. Murray AM, McDonald SE, Archbold P, et al. Cost description of inpatient treatment for ankle fracture. Injury. 2011;42(11):1226-9.
4. McPhail SM, Dunstan J, Canning J, et al. Life impact of ankle fractures: qualitative analysis of patient and clinician experiences. BMC musculoskeletal disorders. 2012;13:224.
5. Hedstrom M, Ahl T, Dalen N. Early postoperative ankle exercise. A study of postoperative lateral malleolar fractures. Clin Orthop Relat Res. 1994(300):193-6.
6. Willett KM, Gray B, Moran CG, et al. Orthopaedic trauma research priority-setting exercise and development of a research network. . Injury. 2010;41(7):763-7.
7. Lin C, Donkers N, Refshauge K, et al Rehabilitation for ankle fractures in adults. Cochrane Database of Systematic Reviews. 2012(11).
8. Miguel A Fernandez, Laura Arnel, Jenny Gould et al. Research priorities in fragility fractures of the lower limb and pelvis: a UK priority setting partnership with the James Lind Alliance. BMJ Open . 2018 (8)
9. Willett K, Keene DJ, Mistry D, N et al. Close Contact Casting vs Surgery for Initial Treatment of Unstable Ankle Fractures in Older Adults: A Randomized Clinical Trial. JAMA. 2016;316(14):1455-63.
10. Lamb SE, Marsh JL, Hutton JL, et al, Collaborative Ankle Support T. Mechanical supports for acute, severe ankle sprain: a pragmatic, multicentre, randomised controlled trial. Lancet. 2009;373(9663):575-81.
11. Olerud C, Molander H. A scoring scale for symptom evaluation after ankle fracture. Arch Orthop Trauma Surg. 1984;103(3):190-4.
12. Herdman M, Gudex C, Lloyd A, J, et al. Development and preliminary testing of the new five-level version of EQ-5D (EQ-5D-5L). Qual Life Res. 2011;20(10):1727-36.
13. Salen BA, Spangfort EV, Nygren AL, et al. The Disability Rating Index: an instrument for the assessment of disability in clinical settings. J Clin Epidemiol. 1994;47(12):1423-35.

For peer review only - http://bmjopen.bmj.com/site/about/guidelines.xhtml

527 14. Berntsen GK, Fonnebo V, Tollan A, et al. Forearm bone mineral density by age in 7,620 men and women: the Tromso study, a population-based study. American journal of epidemiology. 2001;153(5):465-73.

530 15. NICE. Guide to the methods of technology appraisal 2013. London: National Institute of Health and Care Excellence. 2013.

532 16. Oppe M, Rand-Hendriksen K, Shah K, et alEuroQol Protocols for Time Trade-Off Valuation of Health Outcomes. Pharmacoeconomics. 2016;34(10):993-1004.

For peer review only

Reporting checklist for protocol of a clinical trial.

Based on the SPIRIT guidelines.

Instructions to authors

Complete this checklist by entering the page numbers from your manuscript where readers will find each of the items listed below.

Your article may not currently address all the items on the checklist. Please modify your text to include the missing information. If you are certain that an item does not apply, please write "n/a" and provide a short explanation.

Upload your completed checklist as an extra file when you submit to a journal.

In your methods section, say that you used the SPIRIT reporting guidelines, and cite them as:

Chan A-W, Tetzlaff JM, Altman DG, Laupacis A, Gøtzsche PC, Krleža-Jerić K, Hróbjartsson A, Mann H, Dickersin K, Berlin J, Doré C, Parulekar W, Summerskill W, Groves T, Schulz K, Sox H, Rockhold FW, Rennie D, Moher D. SPIRIT 2013 Statement: Defining standard protocol items for clinical trials. Ann Intern Med. 2013;158(3):200-207

		Reporting Item	Page Number
Title	#1	Descriptive title identifying the study design, population, interventions, and, if applicable, trial acronym	1
Trial registration	#2a	Trial identifier and registry name. If not yet registered, name of intended registry	1 and 13

For peer review only

Trial registration: data set	#2b	All items from the World Health Organization Trial Registration Data Set	1 and 13
Protocol version	#3	Date and version identifier	1 and 13
Funding	#4	Sources and types of financial, material, and other support	1 and 14
Roles and responsibilities: contributorship	#5a	Names, affiliations, and roles of protocol contributors	14
Roles and responsibilities: sponsor contact information	#5b	Name and contact information for the trial sponsor	9
Roles and responsibilities: sponsor and funder	#5c	Role of study sponsor and funders, if any, in study design; collection, management, analysis, and interpretation of data; writing of the report; and the decision to submit the report for publication, including whether they will have ultimate authority over any of these activities	14
Roles and responsibilities: committees	#5d	Composition, roles, and responsibilities of the coordinating centre, steering committee, endpoint adjudication committee, data management team, and other individuals or groups overseeing the trial,	9 and 10

For peer review only

		if applicable (see Item 21a for data monitoring committee)	
Background and rationale	#6a	Description of research question and justification for undertaking the trial, including summary of relevant studies (published and unpublished) examining benefits and harms for each intervention	2 and 3
Background and rationale: choice of comparators	#6b	Explanation for choice of comparators	7 and 8
Objectives	#7	Specific objectives or hypotheses	3 and 4
Trial design	#8	Description of trial design including type of trial (eg, parallel group, crossover, factorial, single group), allocation ratio, and framework (eg, superiority, equivalence, non-inferiority, exploratory)	4
Study setting	#9	Description of study settings (eg, community clinic, academic hospital) and list of countries where data will be collected. Reference to where list of study sites can be obtained	6
Eligibility criteria	#10	Inclusion and exclusion criteria for participants. If applicable, eligibility criteria for study centres and individuals who will perform the interventions (eg, surgeons, psychotherapists)	6

For peer review only

Interventions: description	#11a	Interventions for each group with sufficient detail to allow replication, including how and when they will be administered	7 and 8
Interventions: modifications	#11b	Criteria for discontinuing or modifying allocated interventions for a given trial participant (eg, drug dose change in response to harms, participant request, or improving / worsening disease)	N/A
Interventions: adherance	#11c	Strategies to improve adherence to intervention protocols, and any procedures for monitoring adherence (eg, drug tablet return; laboratory tests)	7 and 8
Interventions: concomitant care	#11d	Relevant concomitant care and interventions that are permitted or prohibited during the trial	8
Outcomes	#12	Primary, secondary, and other outcomes, including the specific measurement variable (eg, systolic blood pressure), analysis metric (eg, change from baseline, final value, time to event), method of aggregation (eg, median, proportion), and time point for each outcome. Explanation of the clinical relevance of chosen efficacy and harm outcomes is strongly recommended	5 and 6
Participant timeline	#13	Time schedule of enrolment, interventions (including any run-ins and washouts), assessments, and visits for participants. A schematic diagram is highly recommended (see Figure)	5 and 6

For peer review only

Sample size	#14	Estimated number of participants needed to achieve study objectives and how it was determined, including clinical and statistical assumptions supporting any sample size calculations	4
Recruitment	#15	Strategies for achieving adequate participant enrolment to reach target sample size	6
Allocation: sequence generation	#16a	Method of generating the allocation sequence (eg, computer-generated random numbers), and list of any factors for stratification. To reduce predictability of a random sequence, details of any planned restriction (eg, blocking) should be provided in a separate document that is unavailable to those who enrol participants or assign interventions	7
Allocation concealment mechanism	#16b	Mechanism of implementing the allocation sequence (eg, central telephone; sequentially numbered, opaque, sealed envelopes), describing any steps to conceal the sequence until interventions are assigned	7
Allocation: implementation	#16c	Who will generate the allocation sequence, who will enrol participants, and who will assign participants to interventions	7
Blinding (masking)	#17a	Who will be blinded after assignment to interventions (eg, trial participants, care providers, outcome assessors, data analysts), and how	8 and 9

For peer review only

Blinding (masking): emergency unblinding	#17b	If blinded, circumstances under which unblinding is permissible, and procedure for revealing a participant's allocated intervention during the trial	N/A<br><br>No blinding in this study
Data collection plan	#18a	Plans for assessment and collection of outcome, baseline, and other trial data, including any related processes to promote data quality (eg, duplicate measurements, training of assessors) and a description of study instruments (eg, questionnaires, laboratory tests) along with their reliability and validity, if known. Reference to where data collection forms can be found, if not in the protocol	5 and 6
Data collection plan: retention	#18b	Plans to promote participant retention and complete follow-up, including list of any outcome data to be collected for participants who discontinue or deviate from intervention protocols	5, 6 and 9
Data management	#19	Plans for data entry, coding, security, and storage, including any related processes to promote data quality (eg, double data entry; range checks for data values). Reference to where details of data management procedures can be found, if not in the protocol	3
Statistics: outcomes	#20a	Statistical methods for analysing primary and secondary outcomes. Reference to where other	10 and 11

For peer review only

		details of the statistical analysis plan can be found, if not in the protocol	
Statistics: additional analyses	#20b	Methods for any additional analyses (eg, subgroup and adjusted analyses)	10 and 11
Statistics: analysis population and missing data	#20c	Definition of analysis population relating to protocol non-adherence (eg, as randomised analysis), and any statistical methods to handle missing data (eg, multiple imputation)	10 and 11
Data monitoring: formal committee	#21a	Composition of data monitoring committee (DMC); summary of its role and reporting structure; statement of whether it is independent from the sponsor and competing interests; and reference to where further details about its charter can be found, if not in the protocol. Alternatively, an explanation of why a DMC is not needed	10
Data monitoring: interim analysis	#21b	Description of any interim analyses and stopping guidelines, including who will have access to these interim results and make the final decision to terminate the trial	10
Harms	#22	Plans for collecting, assessing, reporting, and managing solicited and spontaneously reported adverse events and other unintended effects of trial interventions or trial conduct	9

For peer review only

Auditing	#23	Frequency and procedures for auditing trial conduct, if any, and whether the process will be independent from investigators and the sponsor	10
Research ethics approval	#24	Plans for seeking research ethics committee / institutional review board (REC / IRB) approval	1 and 13
Protocol amendments	#25	Plans for communicating important protocol modifications (eg, changes to eligibility criteria, outcomes, analyses) to relevant parties (eg, investigators, REC / IRBs, trial participants, trial registries, journals, regulators)	9 and 10
Consent or assent	#26a	Who will obtain informed consent or assent from potential trial participants or authorised surrogates, and how (see Item 32)	6 and 7
Consent or assent: ancillary studies	#26b	Additional consent provisions for collection and use of participant data and biological specimens in ancillary studies, if applicable	7
Confidentiality	#27	How personal information about potential and enrolled participants will be collected, shared, and maintained in order to protect confidentiality before, during, and after the trial	3
Declaration of interests	#28	Financial and other competing interests for principal investigators for the overall trial and each study site	14

For peer review only

Data access	#29	Statement of who will have access to the final trial dataset, and disclosure of contractual agreements that limit such access for investigators	14
Ancillary and post trial care	#30	Provisions, if any, for ancillary and post-trial care, and for compensation to those who suffer harm from trial participation	9 (Directed through sponsor)
Dissemination policy: trial results	#31a	Plans for investigators and sponsor to communicate trial results to participants, healthcare professionals, the public, and other relevant groups (eg, via publication, reporting in results databases, or other data sharing arrangements), including any publication restrictions	13
Dissemination policy: authorship	#31b	Authorship eligibility guidelines and any intended use of professional writers	14
Dissemination policy: reproducible research	#31c	Plans, if any, for granting public access to the full protocol, participant-level dataset, and statistical code	13
Informed consent materials	#32	Model consent form and other related documentation given to participants and authorised surrogates	13
Biological specimens	#33	Plans for collection, laboratory evaluation, and storage of biological specimens for genetic or	N/A

For peer review only

	molecular analysis in the current trial and for future use in ancillary studies, if applicable	No biological specimens taken as part of this protocol

The SPIRIT checklist is distributed under the terms of the Creative Commons Attribution License CC-BY-ND 3.0. This checklist can be completed online using https://www.goodreports.org/, a tool made by the EQUATOR Network in collaboration with Penelope.ai

For peer review only

BMJ Open

Cast versus functional brace in the rehabilitation of patients treated for an ankle fracture: Protocol for the UK study of ankle injury rehabilitation (AIR) multi-centre randomised trial

Journal:	*BMJ Open*
Manuscript ID	bmjopen-2018-027242.R1
Article Type:	Protocol
Date Submitted by the Author:	30-Oct-2018
Complete List of Authors:	Kearney, Rebecca; University of Warwick, Warwick Medical School<br>McKeown, Rebecca; University of Warwick, Warwick Medical School<br>Stevens, Siobhan; University of Warwick, Warwick Medical School<br>Parsons, Nicholas; University of Warwick, Warwick Medical School<br>Parsons, Helen; University of Warwick, Warwick Medical School<br>Wells, Philip; University of Warwick, Warwick Medical School<br>Brown, Jaclyn; University of Warwick, Warwick Medical School<br>Underwood, Martin; University of Warwick, Warwick Medical School<br>Redmond, Anthony; University of Leeds, Leeds Institute of Rheumatology and Musculoskeletal Medicine<br>Mason, James; University of Warwick, Warwick Medical School<br>Costa, Matthew; University of Oxford, Nuffield Department of Orthopaedics, Rheumatology, and Musculoskeletal Sciences
**Primary Subject Heading**:	Rehabilitation medicine
Secondary Subject Heading:	Rehabilitation medicine, Research methods
Keywords:	Rehabilitation medicine < INTERNAL MEDICINE, Foot & ankle < ORTHOPAEDIC & TRAUMA SURGERY, Adult orthopaedics < ORTHOPAEDIC & TRAUMA SURGERY

SCHOLARONE™
Manuscripts

TITLE PAGE

Title

Cast versus functional brace in the rehabilitation of patients treated for an ankle fracture: Protocol for the UK study of ankle injury rehabilitation (AIR) multi-centre randomised trial

Corresponding Author

1. Rebecca S Kearney: Associate Professor, University of Warwick, Warwick Medical School, Gibbet Hill Road, Coventry, CV4 7AL; R.S.Kearney@warwick.ac.uk; 02476 573156

Co-authors

2. Rebecca McKeown: Trial Manager, University of Warwick, Warwick Medical School, Gibbet Hill Road, Coventry, CV4 7AL (R.McKeown.1@warwick.ac.uk)
3. Siobhan Stevens: Trial Manager, University of Warwick, Warwick Medical School, Gibbet Hill Road, Coventry, CV4 7AL (Siobhan.Stevens@warwick.ac.uk)
4. Nicholas Parsons: Associate Professor in Medical Statistics, University of Warwick, Warwick Medical School, Gibbet Hill Road, Coventry, CV4 7AL (Nick.Parsons@warwick.ac.uk)
5. Helen Parsons: Senior Research Fellow, University of Warwick, Warwick Medical School, Gibbet Hill Road, Coventry, CV4 7AL (H.Parsons@warwick.ac.uk)
6. Philip Wells: Research Associate, University of Warwick, Warwick Medical School, Gibbet Hill Road, Coventry, CV4 7AL (Philip.Wells@warwick.ac.uk)
7. Jaclyn Brown: Senior Project Manager, University of Warwick, Warwick Medical School, Gibbet Hill Road, Coventry, CV4 7AL (J.Brown.10.@warwick.ac.uk)
8. Martin Underwood: Professor, University of Warwick, Warwick Medical School, Gibbet Hill Road, Coventry, CV4 7AL (M.Underwood@warwick.ac.uk)
9. Anthony C Redmond: Professor of Clinical Biomechanics, University of Leeds, Leeds Institute for Rheumatic and Musculoskeletal Medicine, Chapel Allerton Hospital, Chapeltown Road, Leeds, LS7 4SA (A.Redmond@leeds.ac.uk)
10. James Mason: Professor, University of Warwick, Warwick Medical School, Gibbet Hill Road, Coventry, CV4 7AL (J.Mason@warwick.ac.uk)
11. Matthew Costa: Professor of Orthopaedic Trauma, Oxford University, Kadoorie Centre, Level 3, John Radcliffe Hospital, Oxford, OX3 9DU, UK (matthew.costa@ndorms.ox.ac.uk)

Keywords: Ankle, fracture, rehabilitation, immobilisation

Word Count: 4,189

For peer review only

For peer review only - http://bmjopen.bmj.com/site/about/guidelines.xhtml

ABSTRACT

Introduction

Each year in the UK over 120,000 people fracture their ankle. It is not known what the best rehabilitation strategy is for these people. Traditionally standard care has involved immobilisation in a plaster cast but an alternative is a functional brace, which can be removed to allow early movement. This paper details the protocol for a multi-centre randomised trial of plaster cast immobilisation versus functional bracing for patients with an ankle fracture.

Methods and analysis

We will recruit adults with a fractured ankle, for which the treating clinician would consider plaster cast to be a reasonable management option. Randomisation will be on a 1:1 basis, stratified by centre, operative or non-operative management and age. Participants will be allocated to either plaster cast or a functional brace, both treatments are widely used. To have 90% power to detect a difference of 10 points on the primary outcome (Olerud and Molander Ankle Score) at the primary outcome time point (16 weeks) we need to randomise a minimum of 478 people. Quality of life and resource use will be collected at six, 10, 16, 24 weeks and 12, 18, 24 months. The differences between treatment groups will be assessed on an intention-to-treat basis. The economic evaluation will adhere to the recommendations of the NICE Reference Case.

Ethics, registration and dissemination

This trial is funded by the National Institute for Health Research commencing 1st January 2017 (NIHR; CDF-2016-09-009). National Research Ethic Committee approved this study on 4th July 2017 (17/WM/0239). It was registered on the International Standard Randomised Controlled Trial Number registry with reference no ISRCTN15537280 on 24th July 2017. The first site opened to recruitment 9th October 2017. The results of this trial will be submitted to a peer-reviewed journal and will inform clinical practice.

ARTICLE SUMMARY: STRENGHTS AND LIMITATIONS OF THIS STUDY

- Broad eligibility criteria to ensure results can be generalised to the wider population.
- UK wide trial across a minimum of 19 centres to optimize external validity
- Primary outcome measure is patient centred.
- Blinding of interventions is not possible.

For peer review only - http://bmjopen.bmj.com/site/about/guidelines.xhtml

MAIN TEXT

Introduction

Nine percent of trauma surgeons' workload in the UK is managing ankle fractures; over 120,000 per year in the UK. A threefold increase is expected by 2030 due to an increase in older adults who are remaining physically active (1, 2). The frequency of this injury is an increasing burden on the NHS year on year (3). The short-term impact of this injury results in physical impairments of pain, stiffness, weakness and swelling. The longer-term impact results in extended time off work and development of posttraumatic arthritis (4, 5).

Management has traditionally included plaster cast immobilisation for several weeks, whilst the bone heals. A cast provides maximum support, however there are potential problems, there are the risks associated with prolonged immobilisation such as muscle atrophy, deep vein thrombosis and joint stiffness. There are the long-term consequences, which include prolonged gait abnormalities, persistent calf muscle weakness and an inability to return to previous activity levels. Functional bracing may address these issues (6). However, it does not provide the same degree of support to the healing bones (5, 7).

A research priority exercise was undertaken by a UK orthopaedic group in 2010 (8). One of the top priority questions was to establish whether there is a functional advantage associated with different rehabilitation plans following an ankle fracture. This was followed by a 2012 Cochrane review (9) which identified the need for further research into the optimal method of immobilisation for both operatively and non-operatively managed acute ankle fractures. This issue was raised again in a James Lind Alliance Priority Setting Partnership in 2018, which included: '*What is the best physiotherapy regime for adults during out-of-hospital recovery from a fragility fracture of the lower limb?*' in its top ten priority research questions (10).

Good Clinical Practice

The trial will be conducted in full conformance with the principles of the Declaration of Helsinki and to Good Clinical Practice guidelines and comply with UK legislation and Warwick Standard Operating Procedures (SOPs). All data will be stored securely and held in accordance with applicable data protection legislation.

CONSORT

For peer review only - http://bmjopen.bmj.com/site/about/guidelines.xhtml

The trial will be reported in line with the CONSORT statement using the non-pharmacological treatment interventions extension.

Aim

Our aim is to determine which of two immobilisation strategies for people with a fractured ankle is superior.

Objectives

Primary Objective

To quantify and draw inferences on observed differences in the Olerud and Molander Ankle Score (OMAS) between functional brace and plaster cast treatment groups sixteen weeks after randomisation.

Secondary Objectives

1) To quantify and draw inferences on observed differences in ankle function assessed using the OMAS score at six weeks, 10 weeks, 24 weeks and 24 months and Manchester-Oxford Foot Questionnaire (MOXFQ) 16 weeks after randomisation.

2) To draw inferences on the observed differences in ankle function assessed using the OMAS scores in operative and non-operative subgroups

3) To draw inferences on the observed differences in ankle function assessed using the OMAS scores in those 50 years of age and over and those 49 years of age and under subgroups

4) To quantify and draw inferences on observed differences in health related quality of life (EQ5D-5) between trial treatment groups at six weeks, 10 weeks, 16 weeks, 24 weeks, 12 months, 18 months and 24 months after randomisation.

5) To quantify and draw inferences on observed differences in Disability Rating Index (DRI) between trial treatment groups at six weeks, 10 weeks, 16 weeks, 24 weeks and 24 months after randomisation.

6) To quantify and draw inferences on observed differences on complication rates between trial treatment groups at six weeks, 10 weeks, 16 weeks, 24 weeks and 24 months after randomisation.

7) To estimate comparative cost-utility of the two trial treatment groups and collect resource use data at six weeks, 10 weeks, 16 weeks, 24 weeks, 12 months, 18 months and 24 months after randomisation.

Methods and analysis

For peer review only - http://bmjopen.bmj.com/site/about/guidelines.xhtml

Study design

This is a UK multi-centre two arm parallel group randomised controlled trial.

Sample size

The primary outcome for this study is the OMAS 16 weeks post injury. The OMAS is measured on a scale between 0 and 100, where higher scores denote better function. We are seeking to show a between group difference of 10 points. This is consistent with the AIM study (11), which set the OMAS equivalence margin between groups to be 6 points. It is also consistent with other similar outcome measures such as the Foot and Ankle Outcome Score (12), and visual analogue pain scores in acute injury; that set MCIDs of approximately 10 points on a 100 point scale in a trial context.

The standard deviation (SD) of the OMAS from previous feasibility work was approximately 28 points. To account for any variation arising from recruiting from multiple study centres, we have selected a conservative estimation of the trial SD of 30 points. The total trial sample size required to detect a difference of 10 points given a SD of 30 points with two-sided significance set at 5% and 90% power is 382 participants. Allowing a margin of 20% loss during follow-up, this gives a minimum figure of 478 participants. If possible, recruiting a larger sample would enable the between group differences of the two groups to be estimated with higher precision. The minimum 382 participants would create a 95% CI of width 8.5 points. If, for example, 625 participants were recruited, this would yield data on around 500 participants at 20% loss to follow up and would enable a 95% confidence interval (CI) of width 7.4 points to be constructed. Given that both interventions are routinely used in clinical practice and are low risk, if recruitment rates are higher than expected then recruitment will continue to a pre-defined end recruitment date to allow greater precision.

Outcome measures

Primary

Olerud and Molander Ankle Score (OMAS) is a self-administered questionnaire consisting of nine different items; pain, stiffness, swelling, stair climbing, running, jumping, squatting, supports and work/activities of daily living (13). These data will be collected at six weeks, 10 weeks, 16 weeks, 24 weeks and 24 months after randomisation.

Secondary

EQ-5D-5L is a validated, generic health-related quality of life measure consisting of five dimensions each with a 5-level response. Each combination of answers is converted into a health utility score

For peer review only

For peer review only - http://bmjopen.bmj.com/site/about/guidelines.xhtml

that has good test-retest reliability, is simple for participants to use, and gives a single preference-based index value for health status that can be used for broader cost-effectiveness comparative purposes (14). These data will be collected at six weeks, 10 weeks, 16 weeks, 24 weeks, 12, 18 and 24 months after randomisation.

MOXFQ is a self-administered questionnaire that consists of 16 items, each with 5 response options comprising 3 separate underlying dimensions; Walking/standing problems (7 items), foot pain (5 items) and issues related to social interaction (4 items). Item responses are each scored from 0 to 4, with 4 representing the most severe state. The scale scores representing each dimension are calculated by summing the responses to each item within that dimension. Raw scale scores are then converted to a metric (0-100; 100=most severe). These data will be collected at 16 weeks only.

DRI is a self-administered questionnaire. It consists of 12 items specifically related to function of the lower limb. The DRI has been proven to be a practical clinical and research instrument, with good responsiveness and acceptability for assessment of disability caused by impairment in the lower limb (15). These data will be collected at 6 weeks, 10 weeks, 16 weeks, 24 weeks and 24 months after randomisation.

Complications will be captured through two mechanisms. Firstly Participants will be asked on each follow up questionnaire collected at 6 weeks, 10 weeks, 16 weeks, 24 weeks, 12, 18 and 24 months after randomisation. Secondly sites will be asked to report complications during the follow up period to the central trial team. All pre-reduction baseline X-Ray/radiographs and also the last X-Ray/radiograph taken before the primary outcome point of 16 weeks will be collected as part of the complication data set.

The primary analysis will concentrate on direct intervention and healthcare/personal social services costs, while wider impact (societal) costs will be included within the sensitivity analyses. Relevant resource use questionnaires will be administered to participants at baseline and all follow-up points, to collect resource use data associated with the interventions under examination. These data will be collected at six weeks, 10 weeks, 16 weeks, 24 weeks, 12, 18 and 24 months after randomisation.

Screening and Eligibility

UK NHS trust sites will be used to screen all adults with a closed ankle fracture (within three weeks of operative management or injury if non-operative) which the treating clinician would consider

For peer review only - http://bmjopen.bmj.com/site/about/guidelines.xhtml

204 plaster cast a reasonable management option will be screened by clinical care. Screening logs will be collected to assess the main reasons for exclusion as well as number of people unwilling to take part.

206

207 *Inclusion criteria*

208 1. Able to give written informed consent
209 2. Aged 18 years or over
210 3. A closed ankle fracture for which the treating clinician would consider plaster cast a reasonable management option
212 4. Randomised within three weeks of operative management or injury if non-operative

213

214 *Exclusion criteria*

215 1. Ankle fracture secondary to known metastatic disease
216 2. Complex intra-articular fracture (e.g. Pilon fracture)
217 3. In the opinion of the surgeon the patient would require manipulation and close contact/moulded casting
219 4. Wound complications contraindicating functional brace intervention
220 5. Known pre-existing neuropathic joint disease contraindicating functional brace intervention
221 6. Previous ankle fracture already randomised in the present trial
222 7. Unable to adhere to trial procedures or complete postal questionnaires

223

224 Consent

225 Potential participants will be provided with verbal and written information about the study. Written informed consent will be obtained by a member of the research team at each site. The right of a potential participant to refuse participation without giving reasons will be respected, and recorded on the screening log. The participant will remain free to withdraw at any time without giving reasons and without prejudice to any further treatment, and will be provided with a contact point where he/she may obtain further information about the trial.

231

232 At the point of initial consent participants will be asked if they consent to be contacted for any future research related to their ankle fracture. Those who consent will be provided with additional verbal and written information about appropriate additional studies related to their ankle fracture.

235

236 Randomisation

For peer review only

Subjects will be randomised in ratio of 1:1 to the two study intervention arms strictly sequentially, as they become registered as eligible for randomisation on a secure password protected web based system. Allocation concealment will be maintained by an independent randomisation team, at the accredited Clinical Trials Unit, who will be responsible for generation of the sequence and will have no role in the allocation of participants.

The treatment group will be allocated by computer using a minimisation algorithm with a random element and stratification by centre, age and operative/non operative management following use of a secure web based randomisation service. The randomisation system will allocate each participant a unique trial number.

Stratification by centre will help ensure that any clustering effect related to the centre itself will be equally distributed in the trial arms. Stratification on the basis of age will be used to ensure that younger participants with normal bone quality sustaining high-energy fractures and older participants with low-energy (fragility) fractures related to osteoporosis are balanced between arms. The age group stratification will be used as a proxy for bone density. Based on previous literature this will be set at those 49 years and under and those 50 years and over (1, 16). A final stratification based on operative/non operative presentation will be implemented.

Post randomisation withdrawals

Unless a participant explicitly withdraws their consent, they will be followed-up and data collected as per the protocol until the end of the trial. For participants explicitly withdrawing consent for follow up procedures, trial data obtained up until the point of withdrawal will be included in the final analysis of the study. Participants will have the option to withdraw from the trial-related questionnaires, but continue to provide routine NHS data for the purposes of the trial e.g. hospital records of subsequent treatment for the ankle fracture. Participants who withdraw will not be replaced. Participants may also be withdrawn from the trial at the discretion of the investigator and/or Trial Steering Committee due to safety concerns.

Interventions

All participants who require ankle fixation will have this performed according to the preferred technique of the operating surgeon, details of the procedure undertaken will be recorded. All participants will then receive normal local care until satisfactory clinical wound check (usually two

For peer review only

weeks post operatively, up to maximum three weeks), at which point the intervention will be applied by a member of the trauma team.

All participants not undergoing surgery will be approached to take part in the trial on first presentation to the trauma team fracture clinic, and will be eligible for trial participation up to a maximum of three weeks from injury. Weight bearing status will be at the discretion of the treating member of the trauma team and recorded on subsequent CRFs.

Control Group - Standard Plaster Cast

Participants will be fitted with cast immobilisation for a minimum of three weeks. It is expected that the control intervention will not exceed eight weeks, however this will be recorded and monitored by the Trial Management group (TMG). The details of the plaster cast material and method of application will be at the discretion of the treating clinician as per local procedures. Once the cast is removed participants will be encouraged to complete active unloaded ankle range of movement exercises, as per routine clinical practice.

Intervention Group - Functional Bracing

Participants will be fitted with a functional brace (A fixed angle design was specified but the brand and manufacturer was at the discretion of the local site) for a minimum of three weeks. It is expected that the intervention will not exceed eight weeks, however this will be recorded and monitored by the TMG. Throughout this period participants will be encouraged to remove their functional brace to complete active unloaded ankle range of movement exercises, little and often as pain allows. An information sheet explaining these exercises will be handed out to all participants randomised to this trial arm.

Rehabilitation

Any other rehabilitation input will be left to the discretion of the treating trauma team member. However, a record of any additional rehabilitation, together with a record of any other interventions will be recorded on follow up CRFs.

Blinding

The participants cannot be blind to their treatment. The treating clinical team cannot be blind either, but will take no part in the assessment of participants.

For peer review only

For peer review only - http://bmjopen.bmj.com/site/about/guidelines.xhtml

All questionnaire data will be collected via phone and postal mechanisms and entered onto the trial central database by a member of the research team, who cannot be blinded to the trial interventions. Data will be presented to the Data monitoring committee (DMC) using a combination of open and closed reports that will be further detailed in the statistical analysis plan (SAP).

Adverse event management

Adverse events will be listed on Case Report Form for return to the 'AIR' central office. Serious adverse events (SAEs) will be entered onto the SAE reporting form and reported to the central study team. All participants experiencing SAEs will be followed-up as per protocol until the end of the trial. All SAEs that occur between date of consent and 24 month follow-up point will be reported to the sponsor (University of Warwick), ethics committee and oversight committees.

Patient and Public Involvement PPI

Patients were consulted to ascertain if the research gaps highlighted by peer reviewed literature were of importance from their perspective. Based on these responses a single site feasibility trial was developed and funded by NIHR (ISRCTN17809322). The feasibility trial team involved two PPI representatives, who continued their roles through to planning, development and delivery of the current main trial. Specifically, they have been active members on the trial management group, reviewed consent procedures and developed patient facing study materials. At the end of the study they will be vital to dissemination plans, which include the development of a lay summary of findings to be distributed through a variety of resources such as the trial website and posted directly to trial participants.

End of trial

The trial will end when all participants have completed their 24-month follow-up. The trial will be stopped prematurely if mandated by the Ethics Committee, following recommendations from relevant oversight committees or funding for the trial ceases. The Research Ethics Committee will be notified in writing within 90 days when the trial has been concluded or within 15 days if terminated early.

Trial oversight

Trial Management Group (TMG)

For peer review only

For peer review only - http://bmjopen.bmj.com/site/about/guidelines.xhtml

The TMG, consisting of the project staff and co-investigators involved in the day-to-day running of the trial, will meet monthly throughout the project. Significant issues will be referred to the Trial Steering Committee (TSC) or Investigators, as appropriate.

Trial Steering Committee (TSC)

The TSC will have an independent chairperson and consist of clinicians, methodologists and lay representation. Meetings will be held not less than once a year. The TSC will take responsibility for approval of the protocol, major decisions (e.g. need to change the protocol)l, monitoring progress of the trial, reviewing relevant information from other sources, considering recommendations from the DMC, informing and advising on all aspects of the trial.

Data Monitoring Committee (DMC)

The DMC will consist of independent members with relevant clinical research and statistical expertise. The DMC meeting frquency will be guided by the DMC chair, but with a plan to be six months into the recruitment phase and regularly thereafter. Confidential reports containing recruitment, protocol compliance, safety data and interim assessments of outcomes will be reviewed by the DMC. The DMC will advise the TSC as to whether there is evidence or reason why any trial procedures should be modified or the study terminated.

Quality Control

Quality assurance checks to ensure integrity of randomisation, study entry procedures and data collection will follow Warwick CTU standard operating procedures.

Statistical analysis plan

Treatment effects will be presented, with appropriate 95% confidence intervals, for both the unadjusted and adjusted analyses. Tests will be two-sided and considered to provide evidence for a significant difference if p-values are less than 0.05 (5% significance level). All analyses will be conducted as intention to treat unless otherwise specified. No interim analyses are planned and interim analyses will be performed only where directed by the DMC, and with the agreement of the TSC.

Baseline data will be summarised to check comparability between treatment arms, and screening data will be checked to highlight any characteristic differences between those individuals in the study, and those eligible but withholding consent. A CONSORT chart illustrating participant flow

For peer review only - http://bmjopen.bmj.com/site/about/guidelines.xhtml

throughout the study will also be produced. Standard statistical summaries will be presented for the primary outcome measure (OMAS) and all secondary outcome measures.

The main analysis will investigate differences in the primary outcome measure, sixteen weeks after randomisation, between the two treatment groups. Unadjusted and adjusted regression analyses will be used to estimate the between group difference. The adjusted analyses will adjust for the stratification variables, baseline scores and any other clinically important variables. More specifically, adjusted mixed-effects modelling will be used where the recruiting centre will be included as a random effect to allow for possible heterogeneity in patient outcomes due to the recruiting centre. Since individual clinicians will treat only a small number of participants enrolled in the trial, we do not expect clinician specific effects to be important in this study and hence will not be modelled. This adjusted mixed-effects linear regression analysis will be reported as the primary analysis, and will be used to assess evidence for differences in outcomes between intervention arms.

Descriptive statistics of patient reported outcome measure (PROM) data (i.e. OMAS, MOXFQ, EQ5D and DRI) at each time point will be calculated with between group analyses following the method set out for the primary analysis above. Patterns of recovery will also be explored. For example, the area-under-the-curve (AUC) for PROM data will be estimated using the trapezoidal rule for each allocation arm

Complications will be summarised with between groups comparisons evaluated using chi-squared tests. Temporal patterns of any complications will be presented graphically and if appropriate, a time-to-event analysis (e.g. Kaplan-Meier survival analysis) will be used to assess the overall risk and risk within individual classes of important complications (e.g. non-union).

Two pre-specified sub-group analyses will be undertaken to assess whether there is evidence that the intervention effect differs between whether the study participants receives operative or non-operative treatment prior to the study intervention and study participants are aged 50 or over at study randomisation.

The subgroup analyses will follow the methods described for the primary analysis, with additional interaction terms incorporated into the mixed-effects regression model to assess the level of support for these hypotheses. The study is not powered to formally test these hypotheses, so they

For peer review only

For peer review only - http://bmjopen.bmj.com/site/about/guidelines.xhtml

will be reported as exploratory analyses only, and as subsidiary to the analysis reporting the main effects of the intervention in the full study population.

Some data may not be available due to voluntary withdrawal of participants, lack of completion of individual data items or general loss to follow-up. Where possible the reasons for data 'missingness' will be ascertained and reported. The nature and pattern of the missingness will be carefully considered, including whether data can be treated as missing completely at random. If judged appropriate, missing data will be imputed using the multiple imputation facilities available in the statistical analysis software.

If imputation is undertaken, the resulting imputed datasets will be analysed, together with appropriate sensitivity analyses. Any imputation methods used for scores and other derived variables will be carefully considered and justified. Reasons for ineligibility, non-compliance, withdrawal or other protocol violations will be stated and any patterns summarised. More formal analysis, for example using logistic regression with 'protocol violation' as a response, may also be appropriate and aid interpretation.

Health economic analysis plan

Prospective economic evaluation, conducted from a NHS and personal social services perspective, will be included. The economic evaluation will estimate the difference in the cost of resource inputs between the two intervention groups, enabling costs and consequences to be compared. The methods will adhere to the recommendations of the NICE Reference Case(17).

Primary research methods will be followed to estimate the costs of the treatment options, including resource inputs associated with the plaster materials and braces, supplementary interventions, adverse events and rehabilitation inputs. Broader resource utilisation associated with the ankle injury will be captured through routine health service data collection systems and participant questionnaires administered at each follow up time point.

Unit costs will be estimated from local and national sources in addition to primary research using established accounting methods. Costs will be standardised to current prices where possible. Health-related quality of life will be measured at the time of consent, and all follow time points using the EQ-5D-5L measure. Responses will be used to generate quality-adjusted life years (QALYs) using the UK time-trade-off (TTO) value set recommended by the EuroQol group (18).

For peer review only - http://bmjopen.bmj.com/site/about/guidelines.xhtml

Within-trial analysis using bivariate regression of costs and QALYs, with multiple imputation of missing data, will inform a probabilistic assessment of incremental treatment cost-effectiveness from a health service perspective. Missingness mechanisms will be explored and multiple imputation methods will be used where appropriate to avoid biases associated with complete case analysis. Costs and outcomes arising after the first year of the trial will be discounted at 3.5%. Sensitivity analyses will be undertaken to explore uncertainty on the incremental cost-effectiveness ratios and to consider issues of generalisability of the study.

More extensive economic modelling using decision-analytic methods may be considered to extend the target population, time horizon and decision context, drawing on best available information from the literature and stakeholder consultations to supplement the trial data. Parameter uncertainty in the decision-analytic model will be explored using probabilistic sensitivity analysis. Longer term costs and consequences will be discounted to present values using discount rates recommended for health technology appraisal in the UK (current discount rate: 3.5%).

Ethics and dissemination

Edgbaston West Midlands Research Ethics Committee approved this study on 4th July 2017 (REC Reference 17/WM/0239). It was registered on the International Standard Randomised Controlled Trial Number registry (ISRCTN15537280) on 24th July 2017. Both functional brace and plaster interventions are currently used across the NHS for the management of ankle fractures. Consequently, both trial interventions reflect current standard practice and do not expose trial participants to any substantial risks over and above standard care currently received.

The results of the trial will be reported first to trial collaborators. The main report will be drafted by the trial coordinating team, and the final version will be agreed by the TSC before submission for publication, on behalf of the collaboration. The trial will be reported in accordance with the Consolidated Standards of Reporting Trials (CONSORT) guidelines (www.consort-statement.org).

The results of this project, full protocol and related documentation will be disseminated through the trial website, peer-reviewed journals, conference presentations amongst the orthopaedic and rehabilitation networks, the National Library for Health, policy makers such as NICE, patient specific newsletters and through local mechanisms at all participating centres.

For peer review only - http://bmjopen.bmj.com/site/about/guidelines.xhtml

Author contributions

RSK, MLC, MU and ACR wrote the background section and developed the research question. RSK, MLC, MU, ACR, RM, JB, RM and SS wrote the research methodology and management sections of the protocol. NP, HP and PW wrote the sample size and statistical analysis sections of the protocol. JM wrote the health economic evaluation section of the protocol. All authors reviewed and approved the final manuscript.

Acknowledgements

The research team would like to acknowledge Richard Grant and Karen Keates for their contribution throughout the feasibility and main trial as PPI Representatives.

Funding

This trial was funded by the National Institute for Health Research commencing 1st January 2017, as part of a personal Fellowship to RSK (NIHR; CDF-2016-09-009).

Data Statement

The data sharing plans for the current study will be updated via ISRCTN

Disclaimer

The funder has not been involved in the design of the study. The views expressed are those of the authors and not necessarily those of the NHS, the NIHR or the Department of Health.

Competing interests

RSK is a member of the UK NIHR HTA CET board, NIHR ICA Doctoral panel and NIHR RfPB board. MLC is a member of the UK NIHR HTA General Board. ACR is a member of the NIHR ICA Clinical lectureship/Senior Clinical Lectureship panel and holder of a NIHR Senior Investigator award. RSK,MLC, ACR, NP, HP, PW, have all been award current and previous NIHR research grants.

MU was Chair of the NICE accreditation advisory committee until March 2017 for which he received a fee. He is chief investigator or co-investigator on multiple previous and current research grants from the UK National Institute for Health Research, Arthritis Research UK and is a co-investigator on grants funded by Arthritis Australia and Australian NHMRC. He has received travel expenses for speaking at conferences from the professional organisations hosting the conferences He is a director and shareholder of Clinvivo Ltd that

For peer review only - http://bmjopen.bmj.com/site/about/guidelines.xhtml

provides electronic data collection for health services research. He is part of an academic partnership with Serco Ltd. He is an editor of the NIHR journal series for which he receives a fee.

Provenance and peer review

Not commissioned; peer reviewed for ethical and funding approval for NIHR Fellowship prior to submission.

References

1. Court-Brown CM, Caesar B. Epidemiology of adult fractures: A review. Injury. 2006;37(8):691-7.
2. Kannus P, Palvanen M, Niemi S, Parkkari J, Jarvinen M. Increasing number and incidence of low-trauma ankle fractures in elderly people: Finnish statistics during 1970-2000 and projections for the future. Bone. 2002;31(3):430-3.
3. Murray AM, McDonald SE, Archbold P, Crealey GE. Cost description of inpatient treatment for ankle fracture. Injury. 2011;42(11):1226-9.
4. McPhail SM, Dunstan J, Canning J, Haines TP. Life impact of ankle fractures: qualitative analysis of patient and clinician experiences. BMC musculoskeletal disorders. 2012;13:224.
5. Kearney RS, Parsons N, Mistry D, Young J, Brown J, O'Beirne-Elliman J, et al. A protocol for a feasibility randomised controlled trial to assess the difference between functional bracing and plaster cast for the treatment of ankle fractures. Pilot Feasibility Stud. 2017;3:11.
6. Hedstrom M, Ahl T, Dalen N. Early postoperative ankle exercise. A study of postoperative lateral malleolar fractures. Clin Orthop Relat Res. 1994(300):193-6.
7. Achten J, Parsons NR, Kearney RL, Maia Schlussel M, Liew AS, Dutton S, et al. Cast versus functional brace in the rehabilitation of patients treated non-operatively for a rupture of the Achilles tendon: protocol for the UK study of tendo achilles rehabilitation (UK STAR) multi-centre randomised trial. BMJ Open. 2017;7(10):e019628.
8. Willett KM, Gray B, Moran CG, Giannoudis PV, Pallister I. Orthopaedic trauma research priority-setting exercise and development of a research network. . Injury. 2010;41(7):763-7.
9. Lin C, Donkers N, Refshauge K, Beckenkamp P, Khera K, Moseley A. Rehabilitation for ankle fractures in adults. Cochrane Database of Systematic Reviews. 2012(11).
10. Alliance JL. Broken Bones in Older people - Musculoskeletal Injury: fragility fracture of the lower limb and pelvis: James Lind Alliance; 2018 [Available from: http://www.jla.nihr.ac.uk/priority-setting-partnerships/broken-bones-in-older-people/.
11. Willett K, Keene DJ, Mistry D, Nam J, Tutton E, Handley R, et al. Close Contact Casting vs Surgery for Initial Treatment of Unstable Ankle Fractures in Older Adults: A Randomized Clinical Trial. JAMA. 2016;316(14):1455-63.
12. Lamb SE, Marsh JL, Hutton JL, Nakash R, Cooke MW, Collaborative Ankle Support T. Mechanical supports for acute, severe ankle sprain: a pragmatic, multicentre, randomised controlled trial. Lancet. 2009;373(9663):575-81.

For peer review only

For peer review only - http://bmjopen.bmj.com/site/about/guidelines.xhtml

13. Olerud C, Molander H. A scoring scale for symptom evaluation after ankle fracture. Arch Orthop Trauma Surg. 1984;103(3):190-4.

14. Herdman M, Gudex C, Lloyd A, Janssen M, Kind P, Parkin D, et al. Development and preliminary testing of the new five-level version of EQ-5D (EQ-5D-5L). Qual Life Res. 2011;20(10):1727-36.

15. Salen BA, Spangfort EV, Nygren AL, Nordemar R. The Disability Rating Index: an instrument for the assessment of disability in clinical settings. J Clin Epidemiol. 1994;47(12):1423-35.

16. Berntsen GK, Fonnebo V, Tollan A, Sogaard AJ, Magnus JH. Forearm bone mineral density by age in 7,620 men and women: the Tromso study, a population-based study. American journal of epidemiology. 2001;153(5):465-73.

17. NICE. Guide to the methods of technology appraisal 2013. London: National Institute of Health and Care Excellence. 2013.

18. Oppe M, Rand-Hendriksen K, Shah K, Ramos-Goni JM, Luo N. EuroQol Protocols for Time Trade-Off Valuation of Health Outcomes. Pharmacoeconomics. 2016;34(10):993-1004.

For peer review only

Reporting checklist for protocol of a clinical trial.

Based on the SPIRIT guidelines.

Instructions to authors

Complete this checklist by entering the page numbers from your manuscript where readers will find each of the items listed below.

Your article may not currently address all the items on the checklist. Please modify your text to include the missing information. If you are certain that an item does not apply, please write "n/a" and provide a short explanation.

Upload your completed checklist as an extra file when you submit to a journal.

In your methods section, say that you used the SPIRIT reporting guidelines, and cite them as:

Chan A-W, Tetzlaff JM, Altman DG, Laupacis A, Gøtzsche PC, Krleža-Jerić K, Hróbjartsson A, Mann H, Dickersin K, Berlin J, Doré C, Parulekar W, Summerskill W, Groves T, Schulz K, Sox H, Rockhold FW, Rennie D, Moher D. SPIRIT 2013 Statement: Defining standard protocol items for clinical trials. Ann Intern Med. 2013;158(3):200-207

		Reporting Item	Page Number
Title	#1	Descriptive title identifying the study design, population, interventions, and, if applicable, trial acronym	1
Trial registration	#2a	Trial identifier and registry name. If not yet registered, name of intended registry	1 and 13

For peer review only

Trial registration: data set	#2b	All items from the World Health Organization Trial Registration Data Set	1 and 13
Protocol version	#3	Date and version identifier	1 and 13
Funding	#4	Sources and types of financial, material, and other support	1 and 14
Roles and responsibilities: contributorship	#5a	Names, affiliations, and roles of protocol contributors	14
Roles and responsibilities: sponsor contact information	#5b	Name and contact information for the trial sponsor	9
Roles and responsibilities: sponsor and funder	#5c	Role of study sponsor and funders, if any, in study design; collection, management, analysis, and interpretation of data; writing of the report; and the decision to submit the report for publication, including whether they will have ultimate authority over any of these activities	14
Roles and responsibilities: committees	#5d	Composition, roles, and responsibilities of the coordinating centre, steering committee, endpoint adjudication committee, data management team, and other individuals or groups overseeing the trial,	9 and 10

For peer review only

		if applicable (see Item 21a for data monitoring committee)	
Background and rationale	#6a	Description of research question and justification for undertaking the trial, including summary of relevant studies (published and unpublished) examining benefits and harms for each intervention	2 and 3
Background and rationale: choice of comparators	#6b	Explanation for choice of comparators	7 and 8
Objectives	#7	Specific objectives or hypotheses	3 and 4
Trial design	#8	Description of trial design including type of trial (eg, parallel group, crossover, factorial, single group), allocation ratio, and framework (eg, superiority, equivalence, non-inferiority, exploratory)	4
Study setting	#9	Description of study settings (eg, community clinic, academic hospital) and list of countries where data will be collected. Reference to where list of study sites can be obtained	6
Eligibility criteria	#10	Inclusion and exclusion criteria for participants. If applicable, eligibility criteria for study centres and individuals who will perform the interventions (eg, surgeons, psychotherapists)	6

For peer review only

Interventions: description	#11a	Interventions for each group with sufficient detail to allow replication, including how and when they will be administered	7 and 8
Interventions: modifications	#11b	Criteria for discontinuing or modifying allocated interventions for a given trial participant (eg, drug dose change in response to harms, participant request, or improving / worsening disease)	N/A
Interventions: adherance	#11c	Strategies to improve adherence to intervention protocols, and any procedures for monitoring adherence (eg, drug tablet return; laboratory tests)	7 and 8
Interventions: concomitant care	#11d	Relevant concomitant care and interventions that are permitted or prohibited during the trial	8
Outcomes	#12	Primary, secondary, and other outcomes, including the specific measurement variable (eg, systolic blood pressure), analysis metric (eg, change from baseline, final value, time to event), method of aggregation (eg, median, proportion), and time point for each outcome. Explanation of the clinical relevance of chosen efficacy and harm outcomes is strongly recommended	5 and 6
Participant timeline	#13	Time schedule of enrolment, interventions (including any run-ins and washouts), assessments, and visits for participants. A schematic diagram is highly recommended (see Figure)	5 and 6

For peer review only

Sample size	#14	Estimated number of participants needed to achieve study objectives and how it was determined, including clinical and statistical assumptions supporting any sample size calculations	4
Recruitment	#15	Strategies for achieving adequate participant enrolment to reach target sample size	6
Allocation: sequence generation	#16a	Method of generating the allocation sequence (eg, computer-generated random numbers), and list of any factors for stratification. To reduce predictability of a random sequence, details of any planned restriction (eg, blocking) should be provided in a separate document that is unavailable to those who enrol participants or assign interventions	7
Allocation concealment mechanism	#16b	Mechanism of implementing the allocation sequence (eg, central telephone; sequentially numbered, opaque, sealed envelopes), describing any steps to conceal the sequence until interventions are assigned	7
Allocation: implementation	#16c	Who will generate the allocation sequence, who will enrol participants, and who will assign participants to interventions	7
Blinding (masking)	#17a	Who will be blinded after assignment to interventions (eg, trial participants, care providers, outcome assessors, data analysts), and how	8 and 9

For peer review only

Blinding (masking): emergency unblinding	#17b	If blinded, circumstances under which unblinding is permissible, and procedure for revealing a participant's allocated intervention during the trial	N/A<br>No blinding in this study
Data collection plan	#18a	Plans for assessment and collection of outcome, baseline, and other trial data, including any related processes to promote data quality (eg, duplicate measurements, training of assessors) and a description of study instruments (eg, questionnaires, laboratory tests) along with their reliability and validity, if known. Reference to where data collection forms can be found, if not in the protocol	5 and 6
Data collection plan: retention	#18b	Plans to promote participant retention and complete follow-up, including list of any outcome data to be collected for participants who discontinue or deviate from intervention protocols	5, 6 and 9
Data management	#19	Plans for data entry, coding, security, and storage, including any related processes to promote data quality (eg, double data entry; range checks for data values). Reference to where details of data management procedures can be found, if not in the protocol	3
Statistics: outcomes	#20a	Statistical methods for analysing primary and secondary outcomes. Reference to where other	10 and 11

For peer review only

		details of the statistical analysis plan can be found, if not in the protocol	
Statistics: additional analyses	#20b	Methods for any additional analyses (eg, subgroup and adjusted analyses)	10 and 11
Statistics: analysis population and missing data	#20c	Definition of analysis population relating to protocol non-adherence (eg, as randomised analysis), and any statistical methods to handle missing data (eg, multiple imputation)	10 and 11
Data monitoring: formal committee	#21a	Composition of data monitoring committee (DMC); summary of its role and reporting structure; statement of whether it is independent from the sponsor and competing interests; and reference to where further details about its charter can be found, if not in the protocol. Alternatively, an explanation of why a DMC is not needed	10
Data monitoring: interim analysis	#21b	Description of any interim analyses and stopping guidelines, including who will have access to these interim results and make the final decision to terminate the trial	10
Harms	#22	Plans for collecting, assessing, reporting, and managing solicited and spontaneously reported adverse events and other unintended effects of trial interventions or trial conduct	9

For peer review only

Auditing	#23	Frequency and procedures for auditing trial conduct, if any, and whether the process will be independent from investigators and the sponsor	10
Research ethics approval	#24	Plans for seeking research ethics committee / institutional review board (REC / IRB) approval	1 and 13
Protocol amendments	#25	Plans for communicating important protocol modifications (eg, changes to eligibility criteria, outcomes, analyses) to relevant parties (eg, investigators, REC / IRBs, trial participants, trial registries, journals, regulators)	9 and 10
Consent or assent	#26a	Who will obtain informed consent or assent from potential trial participants or authorised surrogates, and how (see Item 32)	6 and 7
Consent or assent: ancillary studies	#26b	Additional consent provisions for collection and use of participant data and biological specimens in ancillary studies, if applicable	7
Confidentiality	#27	How personal information about potential and enrolled participants will be collected, shared, and maintained in order to protect confidentiality before, during, and after the trial	3
Declaration of interests	#28	Financial and other competing interests for principal investigators for the overall trial and each study site	14

For peer review only

Data access	#29	Statement of who will have access to the final trial dataset, and disclosure of contractual agreements that limit such access for investigators	14
Ancillary and post trial care	#30	Provisions, if any, for ancillary and post-trial care, and for compensation to those who suffer harm from trial participation	9 (Directed through sponsor)
Dissemination policy: trial results	#31a	Plans for investigators and sponsor to communicate trial results to participants, healthcare professionals, the public, and other relevant groups (eg, via publication, reporting in results databases, or other data sharing arrangements), including any publication restrictions	13
Dissemination policy: authorship	#31b	Authorship eligibility guidelines and any intended use of professional writers	14
Dissemination policy: reproducible research	#31c	Plans, if any, for granting public access to the full protocol, participant-level dataset, and statistical code	13
Informed consent materials	#32	Model consent form and other related documentation given to participants and authorised surrogates	13
Biological specimens	#33	Plans for collection, laboratory evaluation, and storage of biological specimens for genetic or	N/A

For peer review only

	molecular analysis in the current trial and for future use in ancillary studies, if applicable	No biological specimens taken as part of this protocol

The SPIRIT checklist is distributed under the terms of the Creative Commons Attribution License CC-BY-ND 3.0. This checklist can be completed online using https://www.goodreports.org/, a tool made by the EQUATOR Network in collaboration with Penelope.ai

For peer review only
