# Supplementary material for: Cast versus functional brace in the rehabilitation of patients treated for an ankle fracture: protocol for the UK study of ankle injury rehabilitation (AIR) multicentre randomised trial
Source: BMJ Open. 2018 Dec 18;8(12):e027242. doi: 10.1136/bmjopen-2018-027242 (PMC6303686; doi:10.1136/bmjopen-2018-027242)
Supplement: Reviewer comments [file bmjopen-2018-027242.reviewer_comments.pdf]

PEER REVIEW HISTORY

BMJ Open publishes all reviews undertaken for accepted manuscripts. Reviewers are asked to complete a checklist review form (**http://bmjopen.bmj.com/site/about/resources/checklist.pdf**) and are provided with free text boxes to elaborate on their assessment. These free text comments are reproduced below.

ARTICLE DETAILS

**TITLE (PROVISIONAL)**	Cast versus functional brace in the rehabilitation of patients treated for an ankle fracture: Protocol for the UK study of ankle injury rehabilitation (AIR) multi-centre randomised trial
**AUTHORS**	Kearney, Rebecca; McKeown, Rebecca; Stevens, Siobhan; Parsons, Nicholas; Parsons, Helen; Wells, Philip; Brown, Jaclyn; Underwood, Martin; Redmond, Anthony; Mason, James; Costa, Matthew L

This article was not externally reviewed at BMJ Open. Protocols that have been independently assessed prior to submission to BMJ Open are usually fast-tracked to publication on the grounds that further substantial changes will not be possible. This independent assessment will usually be external, independent review for both a substantial grant award from a non-commercial or government funder and ethics approval.
